# Early Immune Cell and Antibody Kinetics Following SARS-CoV-2 Vaccination in Healthy Adults and Low-Count Monoclonal B-Cell Lymphocytosis

**DOI:** 10.3390/ijms26020681

**Published:** 2025-01-15

**Authors:** Guillermo Oliva-Ariza, Ignacio Criado, Blanca Fuentes-Herrero, Cristina Carbonell, José Ignacio Sánchez-Gallego, Amparo López-Bernús, María Laura Gutiérrez, Alejandro Rolo-Ramírez, Marta Bernal-Ribes, Yolimar Almenara-Morales, Quentin Lecrevisse, Jacques J. M. van Dongen, Miguel Marcos, Julia Almeida, Alberto Orfao

**Affiliations:** 1 Translational and Clinical Research Program, Cancer Research Center (IBMCC, Instituto de Biología Molecular y Celular del Cáncer, CSIC—University of Salamanca), Cytometry Service, NUCLEUS, 37007 Salamanca, Spain; goliva@usal.es (G.O.-A.); ignaciocriado@usal.es (I.C.); blancafh@usal.es (B.F.-H.); joseignaciosanchezgallego@gmail.com (J.I.S.-G.); mlgutierrez@usal.es (M.L.G.); martabernalribes19@gmail.com (M.B.-R.); quentin@usal.es (Q.L.); j.j.m.van_dongen@lumc.nl (J.J.M.v.D.); 2 Department of Medicine, University of Salamanca, 37007 Salamanca, Spain; carbonell@usal.es (C.C.); alopezb@saludcastillayleon.es (A.L.-B.); mmarcos@usal.es (M.M.); 3 Institute of Biomedical Research of Salamanca (IBSAL), 37007 Salamanca, Spain; 4 Department of Internal Medicine, University Hospital of Salamanca, 37007 Salamanca, Spain; rolo.ale@gmail.com (A.R.-R.); alme_real7@hotmail.com (Y.A.-M.); 5 Department of Infectious Diseases, University Hospital of Salamanca, 37007 Salamanca, Spain; 6 Centro de Investigación de Enfermedades Tropicales de la Universidad de Salamanca (CIETUS), 37007 Salamanca, Spain; 7 Biomedical Research Networking Centre Consortium of Oncology (CIBERONC), Instituto de Salud Carlos III, 28029 Madrid, Spain

**Keywords:** Immune response, SARS-CoV-2, COVID-19, vaccination, low-count monoclonal B-cell lymphocytosis, immunodeficiency

## Abstract

The early immune kinetics after SARS-CoV-2 vaccination remain poorly understood, particularly among individuals with low-count monoclonal B-cell lymphocytosis (MBL^lo^). We investigated the cellular and humoral kinetics in the blood of 50 non-MBL healthy donors (HD) vs. 16 MBL^lo^ subjects after SARS-CoV-2 vaccination, who were subclassified according to their history of previous exposure to SARS-CoV-2 into SARS-CoV-2 naïve and previously infected subjects. Overall, we found decreased neutrophil and lymphocyte counts at day +4 following each dose in non-MBL HD, together with an earlier and higher increase in plasma cell (PC) counts and SARS-CoV-2-specific antibody levels after the first vaccine in previously infected non-MBL HD. MBL^lo^ subjects showed a similar profile, except for lower B-cell and higher PC counts after vaccination, and a trend towards a higher (but delayed) antibody response. In summary, we found different cell-kinetic profiles following vaccination in SARS-CoV-2 naïve vs. previously infected non-MBL HD (earlier PC and antibody responses in the latter group); additionally, MBL^lo^ subjects had significantly lower B-cell and higher PC counts after vaccination, and a delayed SARS-CoV-2-specific antibody response.

## 1. Introduction

COVID-19 emerged in December 2019 as a new infectious disease that rapidly spread into a global pandemic [1,2]. From the first reports, it was observed that COVID-19 patients exhibited a heterogeneous clinical course [2,3]. Since then, many reports have highlighted that the severity of the disease is correlated with inadequate innate and adaptive immune responses, associated with an exacerbated inflammatory response, pointing out the importance of an efficient immune response for controlling and completely clearing the virus [4,5,6]. In this context, understanding the immune response to SARS-CoV-2 became crucial for disease management and the development of new vaccination strategies. In an unprecedented global effort, several highly effective vaccines were developed, which prevented severe COVID-19, reducing hospitalizations and deaths related to the virus [7,8,9].

Humoral responses to SARS-CoV-2 are primarily dependent on immunoglobulin (Ig) G (mostly due to IgG1, and to a lesser extent also, the IgG3 antibody subclasses) and IgA vs. IgM-specific antibody isotypes [10,11,12]. After the waning of antibody levels following infection or vaccination, long-lived memory B-cells (MBC) and T-cells become the central immune components for long-term protection [12,13,14]. Despite this, most studies aiming at monitoring the immune system protection acquired after vaccination have mostly focused on the plasma levels of the SARS-CoV-2-specific antibodies generated [10,11,12]. In contrast, the early changes that occur in the immune cell compartments in response to SARS-CoV-2 vaccination of otherwise healthy subjects remain to be investigated in detail.

Studies on SARS-CoV-2 vaccination in the general population have often overlooked factors linked to dysfunctional immune responses during COVID-19, such as low-count monoclonal B-cell lymphocytosis (MBL^lo^) [15,16], a highly prevalent condition (from 4% to 16%) in the general adult population [17,18,19]. Recent studies have shown that MBL^lo^ is associated with secondary humoral (antibody) and B-cell immunodeficiency [20,21,22,23], which makes these subjects more prone to developing more severe infections and secondary tumors [23,24,25]. In the context of SARS-CoV-2 infection, MBL^lo^ has also emerged recently as a novel risk factor for developing more severe COVID-19 [26,27], due to a delayed but more pronounced rise, in the absolute counts of blood circulating plasma cell (PC), compared to non-MBL COVID-19 patients [28]. Although recent studies have reported a defective humoral response to vaccination against SARS-CoV-2 in high-count MBL (MBL^hi^) and CLL patients [29,30], no data have been reported so far regarding the response to vaccination in individuals with MBL^lo^.

Here, we analyzed the kinetics of circulating immune-cell populations, in parallel to the levels of anti-spike SARS-CoV-2-specific antibodies in the blood of healthy donors without MBL (classified according to the existence or not of previous exposure to SARS-CoV-2) the first 30 days after COVID-19 vaccination and compared their response to that found in MBL^lo^ vaccinated subjects.

## 2. Results

### 2.1. Kinetics of the Major Populations of Blood Leukocytes from Non-MBL HD Following Vaccination

**Figure 1:**
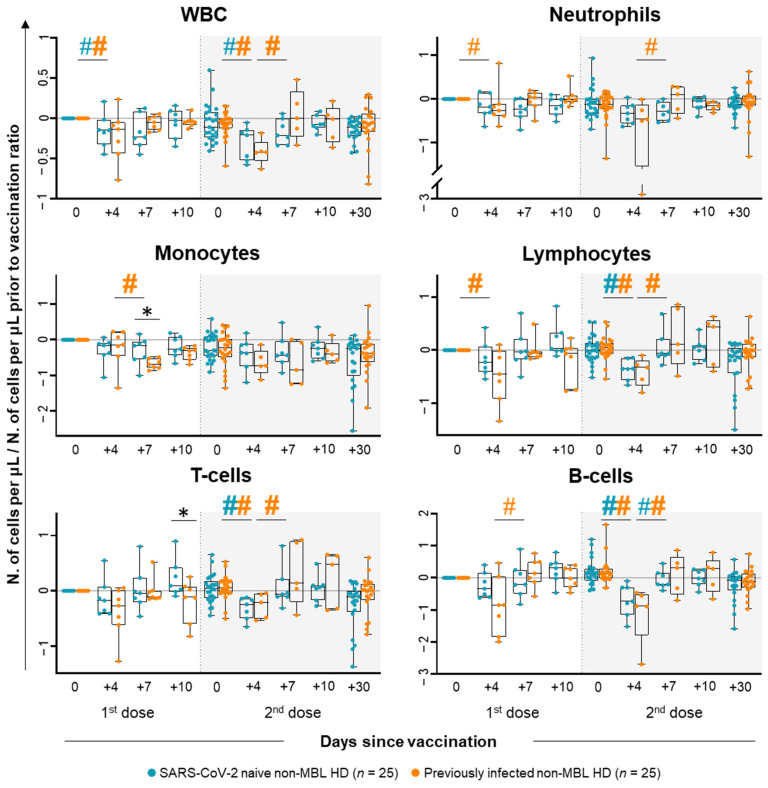
Kinetics of the major populations of leukocytes with a statistically significant different distribution in blood of SARS-CoV-2 naïve vs. previously infected non-MBL HD following SARS-CoV-2 vaccination. Data expressed as the ratio (considering 0 as no change vs. pre-vaccination time point) between the absolute cell count/μL of each cell population and the cell count detected for the same cell population at baseline, prior to vaccination. Subjects without previous contact with SARS-CoV-2 (*n* = 25; blue dots) and those who had been previously infected by the virus (*n* = 25; orange dots) are grouped according to the number of days from the administration of the vaccine. Notched boxes represent 25th and 75th percentile values (IQR), whereas the line in the middle corresponds to median values, and whiskers represent the maximum and minimum values observed for each group. * Statistically significant differences (*p* ≤ 0.05) between SARS-CoV-2 naïve vs. previously infected non-MBL HD; # Statistically significant differences (*p* ≤ 0.05) between the time point analyzed and the previous one for each (color-coded) group of individuals. Hashtags (#) depicted in bold refer to statistically significant differences when considering (more stringent) FDR (<5% vs. <10%) for multiple comparisons. Abbreviations: HD, healthy donors; IQR, interquartile range; MBL, monoclonal B-cell lymphocytosis; WBC, white blood cells.

After the administration of the first dose of the vaccine, a decrease in the absolute white blood cell (WBC) count (vs. pre-vaccination levels) was observed at day +4 following the first dose among non-MBL HD, with a 0.15-fold decrease for SARS-CoV-2 naïve and a 0.13-fold decrease for previously infected subjects, respectively (Figure 1). Among previously infected non-MBL HD, such WBC decrease at day +4 post-vaccination was due to the reduction in the total neutrophil (0.27-fold decrease), and lymphocyte counts (0.45-fold decrease), particularly due to lower NK-cell counts (0.93-fold decrease) (Figure 1 and Supplementary Figure S1). Though no overall differences in the WBC count were observed in any of the two groups at days +7 and +10 after the first injection of the vaccine, non-MBL HD who had previously been infected with SARS-CoV-2 showed a transient reduction in monocyte counts (0.68-fold vs. 0.14-fold decrease at day +4), paralleled by an increase in total B cells at day +7 (0.85-fold decrease at day +4 vs. 0.14-fold increase at day +7) (Figure 1). Despite the kinetics for the major WBC subsets observed after the first dose of the vaccine showed similar profiles in both SARS-CoV-2 naïve and previously infected non-MBL HD—slight but statistically significant differences were found between them. Thus, previously infected non-MBL HD showed markedly lower monocyte counts at day +7 (0.68-fold decrease vs. 0.16-fold decrease in SARS-CoV-2 naïve non-MBL HD) and total T-cell counts at day +10 (0.12-fold decrease vs. 0.09-fold increase in SARS-CoV-2 naïve non-MBL HD) (Figure 1). 

**Figure 2:**
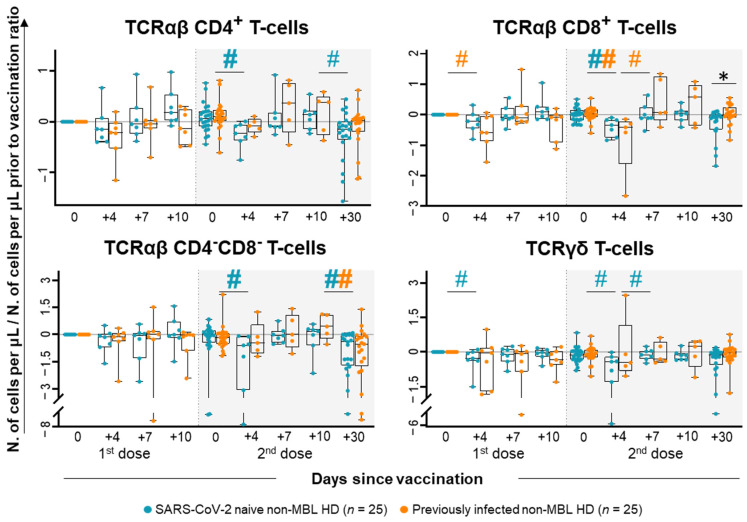
T-cell subset kinetics in the blood of SARS-CoV-2 naïve vs. previously infected non-MBL HD following SARS-CoV-2 vaccination. Data expressed as the ratio (considering 0 as no change vs. pre-vaccination time point) between the absolute cell count/μL of each cell population and the cell count detected for the same cell population at baseline, prior to vaccination. Subjects without previous contact with SARS-CoV-2 (*n* = 25; blue dots) and those who had been previously infected by the virus (*n* = 25; orange dots) are grouped according to the number of days from the administration of the vaccine. Notched boxes represent 25th and 75th percentile values (IQR), whereas the line in the middle corresponds to median values, and whiskers represent the maximum and minimum values observed for each group. * Statistically significant differences (*p* ≤ 0.05) between SARS-CoV-2 naïve vs. previously infected non-MBL HD; # Statistically significant differences (*p* ≤ 0.05) between the time point analyzed and the previous one for each (color-coded) group of individuals. Hashtags (#) depicted in bold refer to statistically significant differences when considering (more stringent) FDR (<5% vs. <10%) for multiple comparisons. Abbreviations: HD, healthy donors; IQR, interquartile range; MBL, monoclonal B-cell lymphocytosis; TCR, T-cell receptor.

A more detailed analysis of the T-cell compartment revealed a statistically significant decrease in TCRαβ^+^CD8^+^ T-cell counts (0.58-fold decrease) at day +4 following the first dose of the vaccine among previously infected non-MBL HD, while SARS-CoV-2 naïve subjects only showed at this time a significant decrease (vs. pre-vaccination) of TCRγδ T-cell counts (0.29-fold decrease) (Figure 2). In contrast, a similar decrease was found for all major T-cell populations in SARS-CoV-2 naïve non-MBL HD after the administration of the second dose of the vaccine, while TCRαβ^+^CD8^+^ T-cells were the only T-cell population that showed statistically significant decreased counts (0.42-fold decrease at day +4 vs. 0.04-fold increase at day 0) in previously infected non-MBL HD (Figure 2). In addition, an overall tendency towards lower TCRαβ^+^ T-cell counts was found in SARS-CoV-2 naïve non-MBL HD at day +30 after the second dose of the vaccine, which was significantly lower for TCRαβ^+^CD8^+^ T-cell numbers in the blood compared to previously infected non-MBL HD (0.02-fold decrease vs. 0.08-fold increase) (Figure 2).

**Figure 3:**
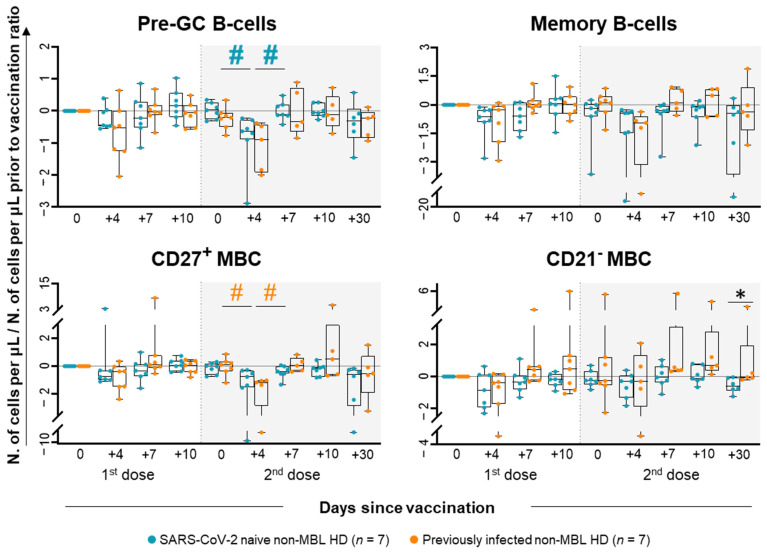
B-cell subset kinetics in blood of SARS-CoV-2 naïve vs. previously infected non-MBL HD following SARS-CoV-2 vaccination. Data expressed as the ratio (considering 0 as no change vs. pre-vaccination time point) between the absolute cell count/μL of each cell population and the cell count detected for the same cell population at baseline, prior to vaccination. Subjects without previous contact with SARS-CoV-2 (*n* = 7; blue dots) and those who had been previously infected by the virus (*n* = 7; orange dots) are grouped according to the number of days from the administration of the vaccine. Notched boxes represent 25th and 75th percentile values (IQR), whereas the line in the middle corresponds to median values, and whiskers represent the maximum and minimum values observed for each group. * Statistically significant differences (*p* ≤ 0.05) between SARS-CoV-2 naïve vs. previously infected non-MBL HD; # Statistically significant differences (*p* ≤ 0.05) between the time point analyzed and the previous one for each (color-coded) group of individuals. Hashtags (#) depicted in bold refer to statistically significant differences when considering (more stringent) FDR (<5% vs. <10%) for multiple comparisons. Abbreviations: GC, germinal center; HD, healthy donors; IQR, interquartile range; MBL, monoclonal B-cell lymphocytosis; MBC, memory B-cells.

When dissecting the B-cell compartment, few differences were observed in the kinetics of the major B-cell subsets throughout the whole follow-up period for any of the two groups of non-MBL subjects (Figure 3 and Supplementary Figures S2 and S3). Despite this, pre-germinal center (GC) (CD19^+^ CD20^+/++^ CD27^−^ IgMD^+^) B-cell counts were significantly decreased in the blood of SARS-CoV-2 naïve non-MBL HD, particularly at day +4 after the second dose of the vaccine, which was followed by a rapid increase at day +7 (0.02-fold increase at day 0 vs. 0.63-fold decrease at day +4 vs. 0.12-fold decrease at day +7) (Figure 3). In previously infected non-MBL HD, CD27^+^ MBC was the only population that transiently decreased at day +4 following the second dose of the vaccine and recovering to normal levels at day +7 (0.10-fold increase at day 0 vs. 1.18-fold decrease at day +4 vs. 0.04-fold increase at day +7). Upon comparing both groups of non-MBL HD, significantly higher CD21- MBC (CD19^+^ CD20^++^ CD5^−^ CD27^−/+^ IgMD^−/+^) counts were found at day +30 after the second dose in blood of previously infected non-MBL HD (0.40-fold increase vs. 0.60-fold decrease in SARS-CoV-2 naïve non-MBL HD) (Figure 3 and Supplementary Figure S2). An in-depth analysis of MBC according to the isotype and subclass of Ig expressed showed that the waning of MBC counts mostly corresponded to fewer IgMD^+^, IgG2^+^ and IgA2^+^ MBC cell counts (Supplementary Figure S3).

**Figure 4:**
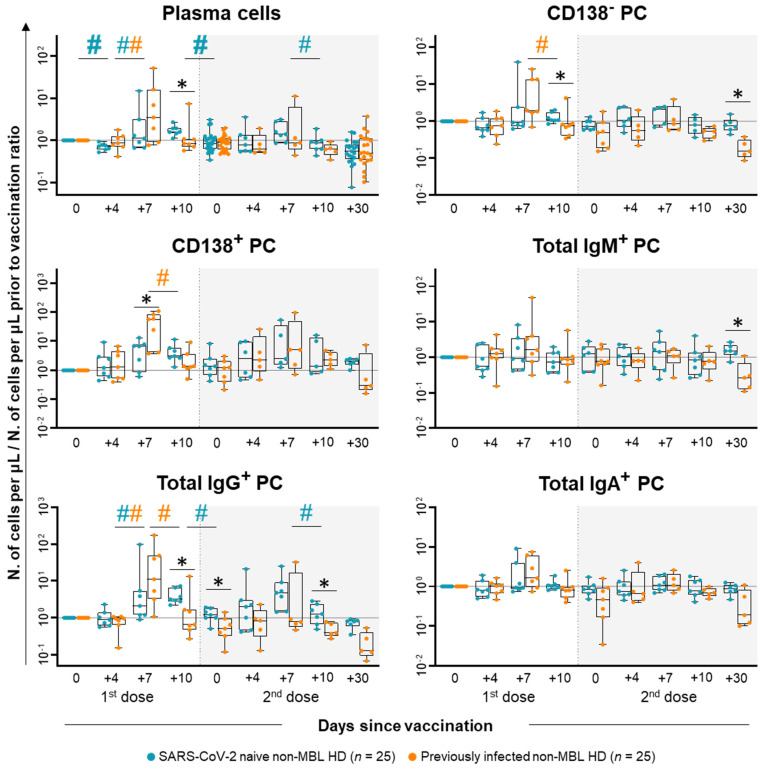
Kinetics of plasma cell subsets in blood grouped by their IgH isotype expression and maturation subsets in SARS-CoV-2 naïve vs. previously infected non-MBL HD following SARS-CoV-2 vaccination. Data expressed as the ratio (considering 0 as no change vs. pre-vaccination time point) between the absolute cell count/μL of each cell population and the cell count detected for the same cell population at baseline, prior to vaccination, using a logarithmic scale. Subjects without previous contact with SARS-CoV-2 (*n* = 25; blue dots) and those who had been previously infected by the virus (*n* = 25; orange dots) are grouped according to the number of days from the administration of the vaccine. Notched boxes represent 25th and 75th percentile values (IQR), whereas the line in the middle corresponds to median values, and whiskers represent the maximum and minimum values observed for each group. * Statistically significant differences (*p* ≤ 0.05) between SARS-CoV-2 naïve vs. previously infected non-MBL HD; # Statistically significant differences (*p* ≤ 0.05) between the time point analyzed and the previous one for each (color-coded) group of individuals. Hashtags (#) depicted in bold refer to statistically significant differences when considering (more stringent) FDR (<5% vs. <10%) for multiple comparisons. Abbreviations: HD, healthy donors; Ig, immunoglobulin; IQR, interquartile range; MBL, monoclonal B-cell lymphocytosis; PC, plasma cells.

In turn, more marked differences were observed between both groups of non-MBL HD in the PC (SSC^int^ FSC^int^ CD45^lo^ CD19^+^ CD20^+/−^ CD38^++^) kinetics in blood, after the administration of the first dose of the vaccine. Thus, total PC counts transiently decreased slightly in the blood of SARS-CoV-2 naïve non-MBL HD at day +4, to progressively increase afterwards with a peak at day +10 (0.38-fold decrease at day +4; 0.11-fold increase at day +7; and 0.59-fold increase at day +10), decreasing (again) thereafter (Figure 4). These kinetics were mainly due to changes in IgG^+^ PC (Figure 4) due to both IgG1^+^ and IgG3^+^ PC (Supplementary Figure S4), while IgM^+^ and IgA^+^ PC counts remained stable over time (Figure 4). However, in previously infected non-MBL HD, IgG^+^ PC counts peaked at day +7, returning to pre-vaccination levels already at day +10 (0.93-fold increase at day +4 vs. 11-fold increase at day +7 vs. 0.65-fold increase at day +10) (Figure 4); similarly to SARS-CoV-2 naïve non-MBL HD, also in previously infected non-MBL HD, IgG1^+^ PCs were the main contributor to these changes (Supplementary Figure S4). As a consequence of the delay in reaching the PC peak in SARS-CoV-2 naïve vs. previously infected subjects, a (low) tendency towards higher total PC and IgG^+^ PC counts were found at day +7 for the latter group, whereas higher total PC and IgG^+^ PC counts, due to both the IgG1^+^ and IgG3^+^ PC counts (Supplementary Figure S4), were observed in the blood of SARS-CoV-2 naïve non-MBL HD at day +10 (Figure 4). Regarding the maturation stage of blood circulating PCs, a statistically significant decline in both the more immature CD138^−^ PC and the mature CD138^+^ PC was observed at day +10 after their peak in the blood of previously infected non-MBL HD. Of note, the number of CD138^−^ PC was significantly higher among SARS-CoV-2 naïve vs. previously infected subjects (1.08-fold vs. 0.75-fold increase) (Figure 4), whilst mature CD138^+^ PC were increased at day +7 after the first dose among previously infected vs. SARS-CoV-2 naïve non-MBL HD (57-fold vs. 6.62-fold increase, respectively) (Figure 4).

In contrast to the PC kinetics observed after the first dose of the vaccine in SARS-CoV-2 naïve non-MBL HD when PC counts reached their peak at day +10, after the second dose, these subjects showed transiently increased PC counts with an earlier peak at day +7 (0.40-fold increase at day +7 vs. 0.13-fold decrease at day +10), which rapidly returned to normal PC levels at day +10. This increase was mostly due to IgG^+^ PC, particularly to IgG1^+^ PC (Figure 4 and Supplementary Figure S4). In turn, previously infected non-MBL HD who had reached the PC peak in the blood at day +7 after the first dose of the vaccine, did not show statistically significant changes in the PC compartment, except for a slight increase in IgG2^+^ PC at day +7 following the second dose of the vaccine (Supplementary Figure S4). These different PC kinetic profiles were associated with overall higher CD138^−^, IgM^+^, and IgG^+^ PC counts (including all IgG subclasses) in the blood of SARS-CoV-2 naïve vs. previously infected non-MBL HD at the later time points investigated (i.e., days +10 and +30 after the second dose) (Figure 4 and Supplementary Figure S4).

Despite all changes described above in the kinetics of immune cells in blood of non-MBL HD following vaccination, the absolute cell counts of the different cell populations remained within the normal (age-matched) ranges throughout the whole follow-up period, except for PC, whose median levels were elevated above the 90th percentile at day +7 (following the first dose of the vaccine) in blood of non-MBL HD who had previously been infected with SARS-CoV-2 (Supplementary Figures S5–S11).

### 2.2. Anti-SARS-CoV-2 Antibody Kinetics in Plasma of Non-MBL HD After Vaccination

**Figure 5:**
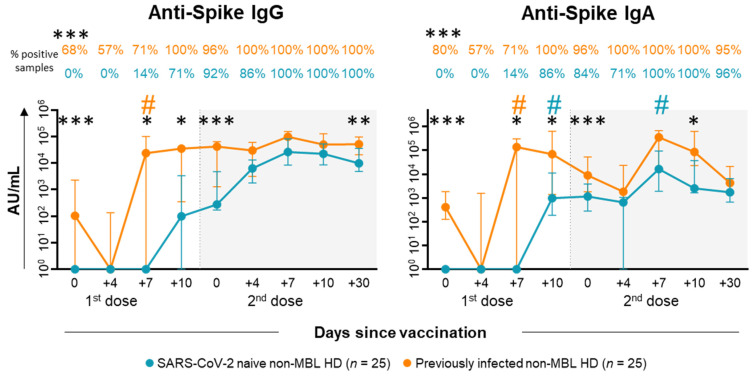
Kinetics of SARS-CoV-2 specific antibody plasma levels in SARS-CoV-2 naïve vs. previously infected non-MBL HD following SARS-CoV-2 vaccination. Plasma levels (AU/mL) of anti-spike SARS-CoV-2 specific IgG and IgA antibodies in SARS-CoV-2 naïve vs. previous COVID-19 non-MBL HD, using a logarithmic scale. Subjects without previous contact with SARS-CoV-2 (*n* = 25; blue dots) and previously infected (*n* = 25; orange dots) are grouped according to the number of days from the administration of the vaccine. Whiskers represent 25th and 75th percentile values (IQR), whereas the point in the middle corresponds to median values. Percentages in the figure panels indicate the proportion of samples with detectable antibodies in plasma. Statistically significant differences between SARS-CoV-2 naïve vs. previously infected non-MBL HD are represented with asterisks (*p*-value: * *p* ≤ 0.05, ** *p* ≤ 0.01, *** *p* ≤ 0.001); # Statistically significant differences (*p* ≤ 0.05) between the time point analyzed and the previous one for each (color-coded) group of individuals. Abbreviations: AU, arbitrary units; HD, healthy donors; Ig, immunoglobulin; IQR, interquartile range; MBL, monoclonal B-cell lymphocytosis.

As regards the humoral response, SARS-CoV-2 naïve non-MBL HD began to seroconvert at day +7 after the first dose of vaccination (Figure 5 and Supplementary Figure S12). Thereafter, both anti-S IgG and IgA antibody levels increased at day +10 after the first dose, with a particularly statistically significant rise for IgA (median [IQR]: 976 [187–11,250] AU/mL at day +10 vs. 0 [0–0] AU/mL at day +7) (Figure 5 and Supplementary Figure S12). Following the administration of the second dose of the vaccine, a similar profile was observed for both antibody isotypes with a peak at day +7, particularly also for the anti-S IgA levels (16,297 [1927–94,617] AU/mL at day +7 vs. 645 [0–1059] AU/mL at day +4) (Figure 5 and Supplementary Figure S12). In those non-MBL HD who had previously been infected withSARS-CoV-2, anti-S IgG and/or IgA antibodies were already detected at baseline in 68% and 80% of cases, respectively (Figure 5 and Supplementary Figure S12). Following the first dose of the vaccine, the titers of both isotypes rapidly increased at day +7 (23,532 [0–100,905] AU/mL at day +7 vs. 0 [0–136] AU/mL at day +4 for IgG); and (136,736 [0–304,759] AU/mL at day +7 vs. 0 [0–1572] AU/mL at day +4 for IgA), all subjects showing a complete seroconversion at day +10 after the first dose of the vaccine (Figure 5 and Supplementary Figure S12). The different kinetic profiles observed in SARS-CoV-2 naïve vs. previously infected non-MBL HD resulted in consistently higher anti-S IgG and IgA antibody titers among individuals from the latter group.

### 2.3. Kinetics of the Major Populations of Blood Leukocytes and Anti-SARS-CoV-2 Plasma Antibodies After Vaccination in MBL^lo^ vs. Non-MBL HD

**Figure 6:**
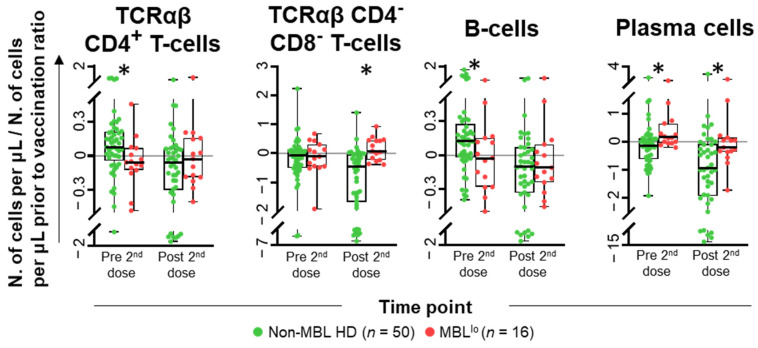
Kinetics of the major populations of leukocytes with a significantly different distribution in the blood of MBL^lo^ vs. non-MBL HD after SARS-CoV-2 vaccination. Data expressed as the ratio (considering 0 as no change vs. pre-vaccination time point) between the absolute cell count/μL of each cell population and the cell count detected for the same cell population at baseline, prior to vaccination. Subjects without MBL (*n* = 50; green dots) and with MBL^lo^ (*n* = 16; red dots) are grouped according to the time from the administration of the vaccine. Notched boxes represent 25th and 75th percentile values (IQR), whereas the line in the middle corresponds to median values, and whiskers represent the maximum and minimum values observed for each group. * Statistically significant differences (*p* ≤ 0.05) between MBL^lo^ vs. non-MBL HD. Abbreviations: HD, healthy donors; IQR, interquartile range; MBL, monoclonal B-cell lymphocytosis; TCR, T-cell receptor.

**Figure 7:**
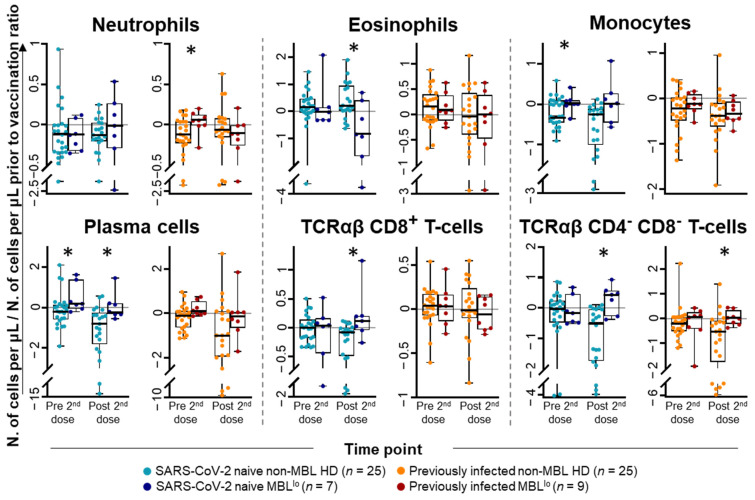
Leukocyte subset kinetics with a significantly different distribution in blood of MBL^lo^ vs. non-MBL HD after SARS-CoV-2 vaccination, grouped according to previous exposure to the virus. Data expressed as the ratio (considering 0 as no change vs. pre-vaccination time point) between the absolute cell count/μL of each cell population and the cell count detected for the same cell population at baseline, prior to vaccination. Subjects without previous contact with SARS-CoV-2 (MBL^lo^, dark blue dots, *n* = 7; non-MBL, light blue dots, *n* = 25) and those who had been previously infected by the virus (MBL^lo^, red dots, *n* = 9; non-MBL, orange dots, *n* = 25) are grouped according to the time from the administration of the vaccine. Notched boxes represent 25th and 75th percentile values (IQR), whereas the line in the middle corresponds to median values, and whiskers represent the maximum and minimum values observed for each group. * Statistically significant differences (*p* ≤ 0.05) between MBL^lo^ vs. non-MBL HD. Abbreviations: HD, healthy donors; IQR, interquartile range; MBL, monoclonal B-cell lymphocytosis; TCR, T-cell receptor.

At baseline (immediately prior to first vaccination), no statistically significant differences were observed in the distribution of the different subsets of blood leukocytes analyzed between MBL^lo^ and non-MBL HD. In contrast, immediately prior to the administration of the second dose of the vaccine, statistically significant differences were found in the distribution of immune cells (vs. pre-vaccination levels) between MBL^lo^ and non-MBL HD. These consisted of lower TCRαβ^+^CD4^+^ T-cell (0.05-fold decrease vs. 0.06-fold increase) and B-cell (0.03-fold decrease vs. 0.11-fold increase) counts, together with higher total PC (0.13-fold increase vs. 0.05-fold decrease) counts in MBL^lo^ vs. non-MBL HD, respectively (Figure 6 and Supplementary Figure S13). Further differences were also observed at day +30 following the second dose of the vaccine, including higher TCRαβ^+^CD4^−^CD8^−^ T-cell (0.06-fold increase vs. 0.49-fold decrease), and PC (0.24-fold decrease vs. 0.96-fold decrease) counts in MBL^lo^ vs. non-MBL HD (Figure 6 and Supplementary Figure S13). These differences between MBL^lo^ and non-MBL HD subjects were due to more prominent differences observed among SARS-CoV-2 naïve individuals, except for the TCRαβ^+^CD4^−^CD8^−^ T-cell counts, which showed higher levels in both SARS-CoV-2 naïve and previously infected subjects (Figure 7 and Supplementary Figures S14 and S15). In addition, significantly higher neutrophil counts (0.06-fold increase vs. 0.12-fold decrease), together with lower eosinophil counts (0.85-fold decrease vs. 0.17-fold increase) were observed in the blood of previously infected MBL^lo^ subjects prior to the second dose of the vaccine, whereas higher TCRαβ^+^CD8^+^ T-cell counts were detected in the blood of SARS-CoV-2 naïve MBL^lo^ subjects at day +30 after vaccination (0.12-fold increase vs. 0.08-fold decrease) (Figure 7 and Supplementary Figures S14 and S15).

In addition to all differences described above in the distribution of immune cells in the blood of MBL^lo^ vs. non-MBL HD prior to the second dose of the vaccine and at day +30 after it, a more detailed longitudinal analysis of the kinetics of the different populations of leukocytes in blood showed similar profiles in both groups of individuals following vaccination throughout the whole follow-up period, except for statistically significant marked higher levels of IgG4+ PC in MBL^lo^ individuals at all time points after the administration of the first dose of the vaccine, together with other slight differences, including transiently increased numbers of monocytes, CD138^−^ PC and IgG2+ PCs were associated with lower levels of IgA2+ PC at specific time points (after the first dose of the vaccine) and higher levels of monocytes with decreased numbers of neutrophils at days +4 and +10 after the second dose in MBL^lo^ vs. non-MBL HD, respectively (Supplementary Figures S16–S20).

**Figure 8:**
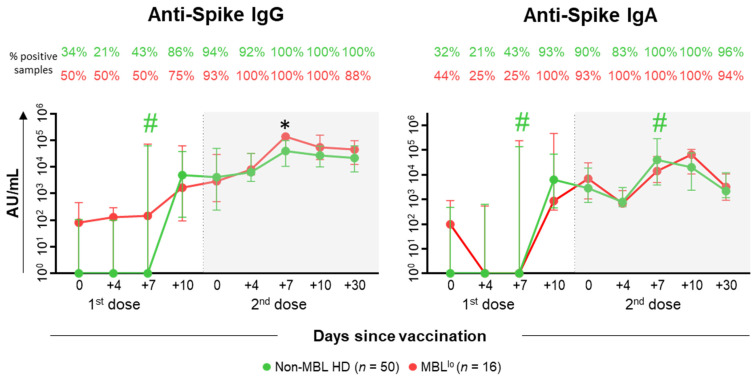
Kinetics of the SARS-CoV-2 specific antibody plasma levels in MBL^lo^ vs. non-MBL HD after SARS-CoV-2 vaccination. Plasma levels (AU/mL) of anti-spike SARS-CoV-2 specific IgG and IgA antibodies in MBL^lo^ vs. non-MBL HD, using a logarithmic scale. Subjects without MBL (*n* = 50; green dots) and with MBL^lo^ (*n* = 16; red dots) are grouped according to the number of days from the administration of the vaccine. Whiskers represent 25th and 75th percentile values (IQR), whereas the point in the middle corresponds to median values. Percentages in the figure panels indicate the proportion of samples with detectable antibodies in plasma. * Statistically significant differences (*p* ≤ 0.05) between MBL^lo^ vs. non-MBL HD. # Statistically significant differences (*p* ≤ 0.05) between the time point analyzed and the previous one for each (color-coded) group of individuals. Abbreviations: AU, arbitrary units; HD, healthy donors; Ig, immunoglobulin; IQR, interquartile range; MBL, monoclonal B-cell lymphocytosis; NK, natural killer; TCR, T-cell receptor.

**Figure 9:**
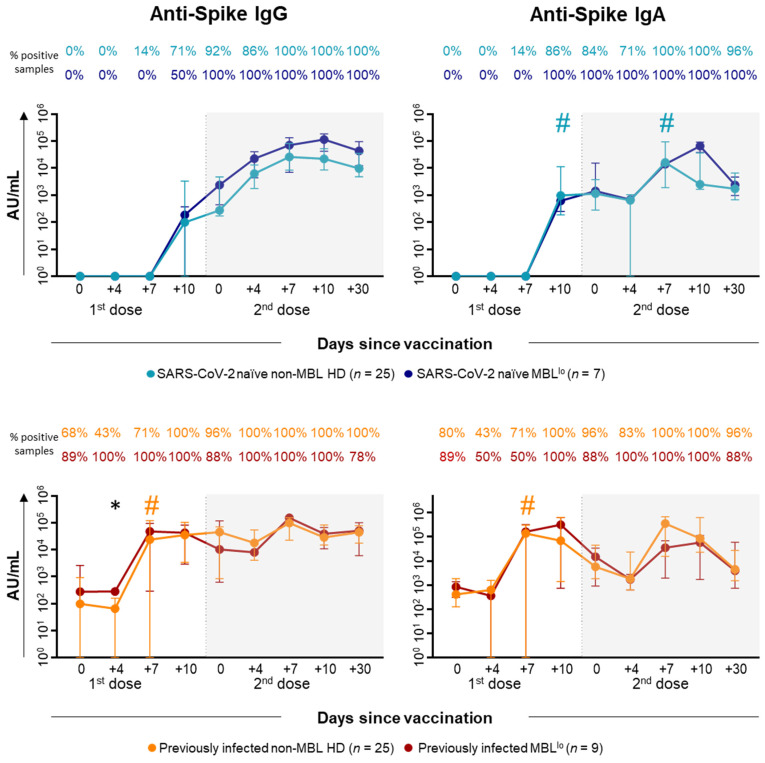
Kinetics of SARS-CoV-2 specific antibody plasma levels in MBL^lo^ vs. non-MBL HD after SARS-CoV-2 vaccination, grouped according to previous exposure to the virus. Plasma levels (AU/mL) of anti-spike SARS-CoV-2 specific IgG and IgA antibodies in MBL^lo^ vs. non-MBL HD, using a logarithmic scale. Subjects without previous contact with SARS-CoV-2 (MBL^lo^, dark blue dots, *n* = 7; non-MBL, light blue dots, *n* = 25) and those who had been previously infected by the virus (MBL^lo^, red dots, *n* = 9; non-MBL, orange dots, *n* = 25) are grouped according to the number of days from the administration of the vaccine. Whiskers represent 25th and 75th percentile values (IQR), whereas the point in the middle corresponds to median values. Percentages in the figure panels indicate the proportion of samples with detectable antibodies in plasma. * Statistically significant differences (*p* ≤ 0.05) between MBL^lo^ vs. non-MBL HD # Statistically significant differences (*p* ≤ 0.05) between the time point analyzed and the previous one for each (color-coded) group of individuals. Abbreviations: AU, arbitrary units; HD, healthy donors; Ig, immunoglobulin; IQR, interquartile range; MBL, monoclonal B-cell lymphocytosis.

Vaccination against SARS-CoV-2 induced a similar humoral response profile in both MBL^lo^ and non-MBL HD (Figure 8). However, when we restricted the analysis to SARS-CoV-2 naïve subjects, slightly superior anti-S IgG levels were observed after the second vaccination among MBL^lo^ vs. non-MBL HD, whereas for those individuals with previous COVID-19 infections, significantly higher anti-S IgG levels were detected at day +4 after the first dose of the vaccine in MBL^lo^ vs. non-MBL HD (281 [259–304] AU/mL vs. 136 [0–183] AU/mL) (Figure 9). Interestingly, among SARS-CoV-2 naïve MBL^lo^ subjects, all antibody levels peaked at day +10 after the second dose of the vaccine, whereas in non-MBL HD they reached their highest levels at day +7 following the second vaccination (Figure 9).

### 2.4. Kinetics of the B-Cell Clones After Vaccination in MBL^lo^ Subjects

The persistence of circulating clonal B-cells throughout the follow-up at similar levels as those detected at baseline was confirmed in all MBL^lo^ individuals. Accordingly, the administration of any of the two doses of the vaccine did not induce statistically significant changes in the size of the MBL clones compared to pre-vaccination levels (median of 0.19 [IQR: 0.08–0.99] clonal B-cells/μL vs. 0.15 [0.05–1.64] clonal B-cells/μL prior to the second dose vs. 0.07 [0.04–1.08] clonal B-cells/μL at day +30 after vaccination; *p* > 0.05) (Supplementary Figure S21).

## 3. Discussion

The introduction of vaccines against SARS-CoV-2 has significantly reduced the number of severe disease cases and deaths related to COVID-19 [7,8,9]. So far, to evaluate the effectiveness of SARS-CoV-2 vaccines, most studies have focused on the humoral response to vaccination through the measurement of the viral-specific antibody responses. The generation of T- and B-cell memory elicited by the vaccine (capable to mount recall responses to viral re-encounter) has been reported in the literature [10,11,12,13,14,31,32,33]. However, few studies have described (integrated data) the early kinetics in the different populations of blood leukocytes in parallel to the humoral changes in response to SARS-CoV-2 vaccination [33,34]. Here, we investigated the kinetics of the immune cell and anti-spike SARS-CoV-2-specific antibodies in the blood of HD (with no evidence of immunodeficiency or under immunomodulatory treatment) in response to their first SARS-CoV-2 vaccination, and compared these kinetics with those observed in a group of individuals carrying small clones of CLL-like B-cells in blood (MBL^lo^), which is known to be associated with an impaired humoral response and higher rates of severe infection [20,21,22,23,25].

Overall, our data show a decrease in WBC counts at day +4 following the administration of the first dose of the vaccine to non-MBL HD, due to decreased neutrophil and lymphocyte counts (mostly due to lower NK cell numbers at this early stage), followed by a decrease in monocytes at day +7, among previously infected non-MBL HD. Similar immune kinetic profiles were observed following the administration of the second dose of the vaccine, including a marked decrease in the blood lymphocyte count at day +4, but now in both SARS-CoV-2 naïve and previously infected non MBL-HD. This transient early decrease in innate cell counts (i.e., neutrophils, monocytes and NK-cells,) in blood, particularly after the first dose of the vaccine might reflect the recruitment of early responders to the site of administration of the vaccine, where priming a local inflammatory response is taking place [35]. However, the relatively limited innate response observed can be explained by the current formulation of mRNA-based vaccines, which contain modified nucleotides to reduce the recognition by immune sensors, such as Toll-like receptors, and thereby restrict the development of an exacerbated inflammatory response as described in the first waves of COVID-19 [36,37]. In addition, we also found decreased counts of both T- and B-cells, particularly at day +4 after the administration of the second dose of the vaccine [33,38]. This might reflect the increased recruitment of these cell populations after the booster vaccination towards the secondary lymphoid tissues for triggering antigen-dependent B-cell activation and maturation in the GC, for the generation of SARS-CoV-2-specific antibody secreting PC and MBC [39]. As regards to T-cell memory, previous studies have demonstrated that SARS-CoV-2 vaccines can elicit both CD4+ and CD8+ memory T-cells, which are detectable for at least 6 months after the second dose of the vaccine [31,40]. Unfortunately, the antibody combinations implemented in our study did not allow us to further characterize the kinetics of specific memory T-cell populations in our cohort. However, since the relative number of both SARS-CoV-2 specific T and B cells against the spike protein are very limited [41], even in subjects with previous exposure to the pathogen, this might explain the lack of a detectable early significant different changes in lymphoid cell subset counts observed in our study after the first dose of the vaccine for previously infected subjects compared to the SARS-CoV-2 naïve group.

As expected, in HD two different profiles of B-cell response to vaccination were observed according to the previous exposure to SARS-CoV-2 status of each individual. Thus, the significant decrease in blood of pre-GC B lymphocytes observed in SARS-CoV-2 naïve non-MBL HD (presumably due to migration to lymph nodes) might reflect a B-cell response dominated by pre-GC B-cells, consistent with the lack of SARS-CoV-2 specific MBC in these individuals [12]. Interestingly, in this group of SARS-CoV-2 naïve HD, a decrease in TCRαβ^+^CD4^+^ T-cells (that collaborate with B-cells in the GC sites) was observed, in parallel to the pre-GC B-cell decrease. In turn, previously infected non-MBL HD showed significantly decreased counts of CD27^+^ MBC in the blood, suggesting a rapid immune reactivation and recruitment of MBC to secondary lymphoid tissues following vaccination in those individuals who had previously been infected with SARS-CoV-2. These data are in line with previous observations by Sokal et al. [42], who described an early predominant expansion of near-germline (extrafollicular) MBC during the first encounter with the pathogen, followed by the expansion of MBC with progressively higher mutated B-cell receptor genes following subsequent contacts with SARS-CoV-2, pointing out the relevance of the refinement of the affinity of the adaptive immune B-cell response for a long-term MBC pool associated with the production of neutralizing antibody secreting cells after reinfection. Of note, an increase in CD21^−^ MBC counts were observed from day +7 after the administration of the second dose of the vaccine both among SARS-CoV-2 naïve and previously infected HD, with a peak at day +10, which was subsequently followed by a decrease toward levels similar to those detected before vaccination. In turn, CD27^−^CD21^−^ MBC counts remained stable in blood throughout the study period, which is in contrast with previous reports in COVID-19 patients describing an expansion of CD27^−^CD21^−^ MBC [42,43].

Previous reports have extensively described a moderate PC response in SARS-CoV-2 naïve individuals at day +10 after the administration of the first dose of the vaccine, followed by an earlier (i.e., day +7) and more intense PC peak after the second dose [34]. In contrast, in previously infected individuals, PC were found to peak at day +7 following the first dose of the vaccine, without a clear PC peak (i.e., response) in blood after the second dose [33]. Our data confirm and extend on these findings, by showing important differences in the PC maturation stages in the blood of non-MBL HD, consisting of an increase in the more mature CD138^+^ PC in both groups of non-MBL HD investigated, particularly among the previously infected non-MBL HD. As previously reported, we also now show that the increased PC counts in blood are due to IgG^+^ PC (mostly IgG1^+^ and, to a lesser extent, also IgG3^+^) while IgM^+^ and IgA^+^ PC remained stable throughout the study [44]. In contrast to the IgG^+^ PC-restricted response found in our study, IgM^+^ and IgA^+^ (in addition to IgG^+^) PC responses have been previously described in COVID-19 [45]. This bias towards a more prominent IgG^+^ PC response found in SARS-CoV-2 vaccination vs. COVID-19, might be due to the intramuscular administration of the vaccine vs. the mucosal entry of the SARS-CoV-2 virus during infection. In line with this hypothesis, low titers of SARS-CoV-2-specific IgA antibodies have been found in the saliva after vaccination [46], suggesting that these kinds of vaccines do not elicit an effective immune response at least at the oral (and probably nasal) mucosa. Furthermore, we found significantly lower levels of most PC subsets in blood of previously infected non-MBL HD at day +30 after the second dose of the vaccine, which might mirror faster homing of PC from blood to bone marrow once the infection has been resolved.

In addition to the immune cell kinetics, we also analyzed the plasma levels of anti-spike SARS-CoV-2-specific IgG and IgA antibodies. Our data show that specific SARS-CoV-2 antibodies had similar kinetics to those described above for PC. Thus, both anti-spike IgG and IgA antibody plasma levels rapidly increased at day +7 after the first dose of the vaccine in previously infected non-MBL HD, when maximum antibody titers were reached. In turn, antibody levels became detectable in most SARS-CoV-2 naïve subjects at day +10 after the first dose of the vaccine, and thereafter they progressively increased with the highest levels being reached at day +7 after the second dose, in line with previous findings [47]. This profile became even more pronounced when we analyzed antibody plasma levels in paired samples from the same individuals during the whole study period. As previously described by other groups [12,34], we also found that anti-spike IgG plasma levels persisted during at least 30 days after the second dose of the vaccine, while IgA plasma levels already started to decline at day +10. Despite the fact that we did not evaluate the neutralizing ability of anti-spike IgG antibodies after each dose of vaccination, several reports have pointed out a (very) high direct correlation between the total and the neutralizing titers of anti-spike IgG antibodies detected after the administration of the vaccine; therefore, it might be expected that a great fraction of the anti-spike IgG antibodies detected here might have virus neutralizing capacity [42,48,49]. Interestingly, Sokal et al. also demonstrated that following natural infection, subsequent contacts with the pathogen shape up the B-cell repertoire from near-germline VDJ rearrangements to a broadly somatic hypermutated B-cell receptor Ig gene profile, with the ability to differentiate to Ig-secreting cells with high neutralization capacity [42]. In the context of vaccination, recent data showed that SARS-CoV-2 vaccines also generate MBC responses with similar levels of somatic hypermutation than those found after the SARS-CoV-2 infection [40,50]. The apparent discrepancy between the rise in anti-spike SARS-CoV-2-specific IgA antibody levels in plasma and IgA^+^ PC counts in blood in the absence of changes in IgA^+^ PC counts in blood following vaccination might indicate that a low number of antigen-specific IgA^+^ PC are enough to produce a significant increase in IgA antibody plasma levels, or that the newly generated IgA^+^ PC are preferentially in the bone marrow and/or mucosa, without a detectable peak in blood. Despite the fact that no data were obtained in our study regarding the immune response to the third dose of the vaccine, previous studies by others have shown that the administration of a third booster 7–9 months after the second dose might result in up to a 35-fold increase in anti-spike IgG-specific antibody titers in plasma, in line with an even stronger humoral response than that observed after the second dose [48,49,51].

MBL^lo^ has been recently identified as a novel biomarker for more severe COVID-19 among otherwise HD [26], potentially due to a delayed and more pronounced PC response, together with significantly higher SARS-CoV-2-specific antibody titers in comparison with non-MBL COVID-19 patients [28]. In addition, other defects in the immune system of non-infected MBL^lo^ subjects have been described, which relate to a defective B-cell compartment and humoral response, associated in these subjects with an increased risk for more severe infections [20,21,22,24,25]. Fully in line with these results, we found lower total B-cell counts, immediately before the administration of the second dose of the vaccine (as previously described for convalescent COVID-19 patients with MBL^lo^) [26] associated with higher PC counts in blood than those observed among non-MBL HD, suggesting also a delayed PC response to vaccination in MBL^lo^ subjects [28]. Of note, these differences were mostly noticeable among SARS-CoV-2 naïve MBL^lo^ subjects, suggesting that individuals with pre-existent memory against SARS-CoV-2 would display a humoral immune response similar to that observed in non-MBL HD. Recent data on MBL^hi^ and CLL patients revealed an impaired antibody response to SARS-CoV-2 vaccination in both groups of patients, associated with the production of lower antibody titers compared to HD (particularly in CLL), leading to the need of additional doses of the vaccine to reach protective antibody levels [29,30]. In contrast, our data on MBL^lo^ subjects show similar anti-spike-specific antibody levels than those reached in non-MBL HD, though our approach does not provide information on the quality of the humoral response against the SARS-CoV-2 vaccine in MBL^lo^ vs. non-MBL individuals (i.e., the neutralizing activity of these antibodies). Despite this, it is worth mentioning that the specific antibody kinetics in MBL^lo^ SARS-CoV-2 naïve subjects revealed a delay in attaining the highest levels of both anti-spike IgG and IgA antibodies until day +10 (vs. day +7 in non-MBL HD) after the second dose of the vaccine, while for SARS-CoV-2 naïve non-MBL HD the peak was observed at day +7, like in non-MBL HD. Of note, such delayed antibody response to reach the highest (antibody) levels was also observed for the IgA antibodies in MBL^lo^ subjects who had been previously infected by SARS-CoV-2, following the first dose of the vaccine. Altogether, our data supports previous observations in MBL^lo^ subjects during COVID-19 [28], where the delay in the peak of anti-SARS-CoV-2 specific antibody levels was also found compared to non-MBL COVID-19 patients, which, in turn, was related to a higher severity of the disease in MBL^lo^ subjects [26].

Interestingly, here we show that the administration of the vaccine against SARS-CoV-2 does not induce an expansion of the clonal B-cells in MBL^lo^ individuals, similar to what has been previously described in COVID-19 patients [26,28]. Altogether, these results support the notion that the exposure to SARS-CoV-2 has not had a significant influence on the size of the MBL^lo^ clone.

To the best of our knowledge, this is the first study in which the early changes in major and minor subsets of WBC in response to SARS-CoV-2 vaccination has been investigated at multiple time points during the first month of administration of the first two doses of the vaccine in parallel to spike-specific IgG and IgA antibody plasma levels, providing a comprehensive overview of the immune cellular and humoral response to SARS-CoV-2 vaccination. The strict inclusion criteria applied to select our cohort reduce the chance for a bias on our results on the immune kinetics in blood, due to the effect of any underlying condition, including MBL^lo^. However, our study has some limitations, related to the relatively small sample size, particularly of MBL^lo^ subjects, which did not allow us to perform a closer longitudinal analysis of the immune cell kinetics in this group of individuals. In addition, our cohort consists of subjects who were under three different vaccination programs, which might contribute to a greater heterogeneity of the results (despite the fact that we did not find statistically significant differences when we compared the different types of vaccines used in our cohort, probably due to the low sample size). Taking into consideration that our study provides no data on antigen-specific B- or T-cell responses, on functional (immune-cell) assays, or on the neutralizing activity of the anti-spike antibodies detected, additional studies are needed to gain insight into the quality (and the real biological impact) of the specific immune responses to SARS-CoV-2 vaccination.

## 4. Materials and Methods

### 4.1. Study Group

A total of 75 adult volunteers (33 men and 42 women; median age of 46 years [IQR: 36–56 years]) who received COVID-19 vaccines between February and April 2021, were included in this study. Subjects were vaccinated with mRNA-based vaccines (BNT162b2, Pfizer-BioNTech or mRNA-1273, Moderna) or adenoviral vector-based vaccine (ChAdOx1 nCoV-19, AstraZeneca), according to the Spanish government vaccination program. Those subjects with an incomplete vaccination schedule (i.e., receiving only one dose of either Pfizer or Moderna vaccines without previous COVID-19 infection) (*n* = 2), individuals presenting active neoplastic disease or a hematologic malignancy (*n* = 1), those with confirmed or suspected acute COVID-19 infection during the period of the study (*n* = 1), or that were under immunomodulatory/immunosuppressive treatment (*n* = 5), were excluded from the study, which resulted in a final cohort of 66 participants. All 66 subjects were screened immediately prior to receiving the first dose of the vaccine (this time point being considered as baseline) for the presence of MBL^lo^, using high-sensitive next-generation flow cytometry (NGF), and subdivided into two groups: (i) MBL^lo^ subjects (16/66 [24%], 7 men and 9 women; median age of 53 years [IQR: 45–65 years]); and (ii) non-MBL HD (50/66 [76%], 21 men and 29 women; median age of 43 years [IQR: 34–51 years]). All subjects included in the study were surveyed for a previous diagnosis of COVID-19; in addition, anti-spike specific antibody levels were measured in plasma of each individual immediately prior to vaccination (i.e., at baseline), to confirm/rule out previous exposure to the pathogen, even in the absence of a prior diagnosis of COVID-19. Subsequently, individuals with MBL^lo^ and non-MBL HD were subclassified according to their history of previous exposure to SARS-CoV-2 into SARS-CoV-2 naïve and previously infected subjects, using the above defined criteria (Supplementary Table S1). As no significant differences were found when groups were compared according to sex or the type of vaccine administered, no further classifications were performed (Supplementary Table S1). A total of 656 pre-pandemic non-MBL HD (301 men and 355 women; median age of 58 years [IQR: 47–71 years]) from the same geographical area, who had been recruited and evaluated for the presence of MBL before the SARS-CoV-2 pandemic, were also included in the study to define the normal ranges for the absolute number of cells for each cell population analyzed.

Each participant gave his/her consent to participate in the study, according to the Declaration of Helsinki and the Spanish law for Biomedical Research. The study and both the subjects’ data and blood samples collected were approved by the Ethics Committee of the University Hospital of Salamanca/IBSAL (approval codes: CEIC PI4705/2017 and PI 2020 03 468).

### 4.2. Immunophenotypic Studies

A total of 295 peripheral blood (PB) samples (71 samples from MBL^lo^ and 224 from non-MBL vaccinated subjects) were collected from the 66 individuals, before the administration of the first dose of the vaccine (defined hereon as baseline), and subsequently before the administration of the second dose of the vaccine and at day +30 after the later had been administered. A subgroup of 18/66 (27%) individuals (4 MBL^lo^ and 14 non-MBL) were enrolled in a closer follow up, where PB samples (36 and 118 samples from the MBL^lo^ and the non-MBL subjects, respectively) were additionally collected at days +4, +7, and +10 after each of the two vaccine doses. All PB samples were stained with the EuroFlow Lymphocyte Screening Tube (LST) and the Immunemonitoring (IMM) BIgH tube, using the EuroFlow bulk-lyse-stain-and-then-fix standard operating procedure [26,52,53] (Supplementary Methods and Supplementary Table S2), available at www.EuroFlow.org (accessed on 20 October 2024). For each antibody combination, ≥107 cells/tube were measured per sample. Automated data analysis was performed using the INFINICYT 2.0.1a software (Cytognos SL, Salamanca, Spain) and the LST and IMM-BIgH data bases [54,55] (Supplementary Methods). The gating strategy used during automated analysis of flow cytometry data and the phenotypic characteristics of the cell populations identified have been previously described in detail for both the LST [54], and the IMM-BIgH tubes (Figure 3 from Delgado et al. [55]) and are also listed (for the major populations) in Supplementary Tables S2B and S2D, respectively.

### 4.3. Measurement of Anti-SARS-CoV-2 IgG and IgA Antibody Plasma Levels

(Semi)quantitative determination of IgG and IgA plasma levels against the spike (S) protein of SARS-CoV-2 was performed in 294 samples, using commercially available ELISA kits (ImmunoStep S.L., Salamanca, Spain) as per the manufacturer’s recommendations.

### 4.4. Statistical Methods

Data were normalized with respect to those values observed at the pre-vaccination time point, by calculating at every follow-up time point, the ratio between the absolute cell count/μL of blood for each cell population, and the cell count detected for the same cell population at baseline, prior to vaccination. Here, we decided to consider 0 as no change vs. the pre-vaccination time point for a clearer interpretation of the results (which means that increments in cell counts are represented with positive values, while decreased cell counts are shown with negative values), by applying the following formulas:

If x ≥ 1; f(x) = x − 1

If x < 1; f(x) = (−1/x) + 1

For the comparison between groups for continuous variables, the Mann–Whitney U or the Kruskal–Wallis non-parametric tests for 2 or >2 independent groups of samples were used, respectively. Corrected (Benjamini–Hochberg procedure) *p* values of ≤0.05 for multiple comparisons with a false discovery rate (FDR) of <10% (or <5% as indicated specifically in the legend of each figure) were used to define statistically significant differences across the different time points. For categorical variables, the Chi-square test and Fisher’s exact test were used; *p* values ≤ 0.05 were considered to be associated with statistical significance. Statistical analyses and figures were carried out and designed using the IBM-SPSS Statistical software v28.0 (IBM, Armonk, NY, USA) and GraphPad Prism V8 software (GraphPad Software, San Diego, CA, USA), respectively.

## 5. Conclusions

In summary, here we found different immune cell kinetic profiles following vaccination in SARS-CoV-2 naïve vs. previously infected non-MBL HD, which consisted of earlier PC and specific antibody specific responses in those non-MBL HD who had undergone COVID-19. The analysis of the immune response to SARS-CoV-2 vaccination in MBL^lo^ subjects revealed an altered immune cell distribution, which mainly affected B-cells and PC, associated with a delayed humoral response to SARS-CoV-2 vaccination in these subjects.

## Data Availability

De-identified data collected during the study on individual patients will be available via the Spanish National DNA Bank Carlos III, immediately after publication, for researchers who provide a proposal that is approved by the external scientific committee and ethics committee of the Spanish National DNA Bank.

## Supplementary Materials

The following supporting information can be downloaded at: https://www.mdpi.com/article/10.3390/ijms26020681/s1. References [56,57] have been mentioned in the text.
