# Supplementary material for: Early Immune Cell and Antibody Kinetics Following SARS-CoV-2 Vaccination in Healthy Adults and Low-Count Monoclonal B-Cell Lymphocytosis
Source: Int J Mol Sci. 2025 Jan 15;26(2):681. doi: 10.3390/ijms26020681 (PMC11765611; doi:10.3390/ijms26020681)
Supplement: Supplementary file 1 [file ijms-26-00681-s001.zip › ijms-3390560-supplementary.pdf]

Supplementary Material

1 Supplementary Materials and Methods

Immunophenotypic studies. Peripheral blood (PB) samples (30mL) were collected in K3-EDTA Vacutainer tubes (Becton/Dickinson Biosciences (BD), San Jose, CA) and immediately (<6 hours) stained with the EuroFlow Lymphocyte Screening Tube (LST) and the Immunemonitoring (IMM) BIgH tube, using the EuroFlow bulk-lyse-stain-and-then-fix standard operating procedure (SOP), available at www.EuroFlow.org.(1,2) Briefly, non-nucleated red blood cells were lysed with an ammonium chloride solution and washed twice in phosphate buffered saline (PBS; pH=7.4). Then, $>10^7$ white blood cells (WBC) were stained for 30 minutes with the LST (BD OneFlow LST^{TM} reagent, BD), and the BIgH, antibody combinations (Supplementary Table 2A and 2C). Stained cells were subsequently fixed and, in case of the IMM-BIgH tube, stained with the cytoplasmic antibodies using the Fix & Perm Reagent Kit (Nordic-MUBio, Susteren, The Netherlands). For each antibody combination, $\geq10^7$ cells/tube were measured per sample, in either a FACSCanto II (BD) or a FACSLyric (BD) flow cytometer for the LST stained aliquot of the sample, and in an LSRFortessa X-20 (BD) or a FACSymphony (BD) instrument, for the EuroFlow IMM-BIgH stained sample aliquot. For data analysis, the Automated Gating and Identification (AGI) tool of the Infinicyt software (Cytognos SL, Salamanca, Spain) was used in combination with the EuroFlow LST and IMM-BIgH databases.(3,4) For each cell population, absolute cell counts/µL of blood were calculated using a dual-platform approach, (5) based on their relative values (% from all blood leukocytes) obtained with the flow cytometer, and the absolute number of leukocytes (i.e., white blood cells) measured in a Sysmex XN-1000TM hematology cell analyzer (Sysmex, Kobe, Japan). For the identification of MBL clones, the presence of ≥20 clustered cells with an aberrant immunophenotype -e.g., $CD5^+$ $CD20^{lo}$ surface membrane immunoglobulin $(smIg)^{lo}$ and/or clonal (e.g. $smIg\kappa^+$ or $smIg\lambda^+$) restricted profile-, was required.(6)

2 Supplementary References

1. Flores-Montero J, Sanoja-Flores L, Paiva B, Puig N, García-Sánchez O, Böttcher S, et al. Next Generation Flow for highly sensitive and standardized detection of minimal residual disease in multiple myeloma. Leukemia. 2017 Oct 1;31(10):2094–103. doi: 10.1038/leu.2017.29

2. Oliva-Ariza G, Fuentes-Herrero B, Carbonell C, Lecrevisse Q, Pérez-Pons A, Torres-Valle A, et al. High frequency of low-count monoclonal B-cell lymphocytosis in hospitalized COVID-19 patients. Blood. 2023;141(3):309–14. doi: 10.1182/blood.2022017439

3. Flores-Montero J, Grigore G, Fluxá R, Hernández J, Fernandez P, Almeida J, et al. EuroFlow Lymphoid Screening Tube (LST) data base for automated identification of blood lymphocyte subsets. J Immunol Methods. 2019 Dec 1;475:112662. doi: 10.1016/j.jim.2019.112662

4. Delgado AH, Fluxa R, Perez-andres M, Diks AM, Brink JAMVG Den, Barkoff A, et al. Automated EuroFlow approach for standardized in-depth dissection of human circulating B-cells and plasma cells. Front Immunol. 2023;14. doi: 10.3389/fimmu.2023.1268686

5. Hultin LE, Chow M, Jamieson BD, O'Gorman MRG, Menendez FA, Borowski L, et al. Comparison of interlaboratory variation in absolute T-cell counts by single-platform and optimized dual-platform methods. Cytom Part B - Clin Cytom. 2010;78(3):194–200. doi: 10.1002/cyto.b.20500

6. Nieto WG, Almeida J, Romero A, Teodosio C, López A, Henriques AF, et al. Increased frequency (12%) of circulating chronic lymphocytic leukemia-like B-cell clones in healthy subjects using a highly sensitive multicolor flow cytometry approach. Blood. 2009;114(2):33–7. doi: 10.1182/blood-2009

7. Blanco E, Pérez-Andrés M, Arriba-Méndez S, Contreras-Sanfeliciano T, Criado I, Pelak O, et al. Age-associated distribution of normal B-cell and plasma cell subsets in peripheral blood. J Allergy Clin Immunol. 2018;141(6):2208-2219.e16. doi: 10.1016/j.jaci.2018.02.017

3 Supplementary Tables

Supplementary Table 1. Baseline clinical and biological characteristics of non-MBL HD and MBL^{lo} donors included in the vaccination study against SARS-CoV-2.

	**SARS-CoV-2 naïve subjects**		**Previously infected subjects**		P-value
	**Non-MBL HD (n=25)**	**MBL^{lo} (n=7)**	**Non-MBL HD (n=25)**	**MBL^{lo} (n=9)**	
**Age (years)***	41 (32–49)	43 (43–46)	43 (36–52)	54 (43–61)	**0.007**[a]
**Sex (male/female)**	11 / 14 (44% / 56%)	4 / 3 (57% / 43%)	10 / 15 (40% / 60%)	3 / 6 (33% / 67%)	0.28
**Type of vaccine**					
ChAdOx1 nCoV-19 (AstraZeneca)	2/25 (8%)	0/7 (0%)	1/25 (4%)	0/9 (0%)	
BNT162b2 (Pfizer-BioNTech)	20/25 (80%)	5/7 (71%)	16/25 (64%)	7/9 (78%)	0.20
mRNA-1273 (Moderna)	3/25 (12%)	2/7 (22%)	8/25 (32%)	2/9 (22%)	

Results expressed as number of cases (percentage) or as * median (interquartile range). [a] P-value when comparing non-MBL HD vs. MBL^{lo} among those cases with previous COVID-19.

Abbreviations (alphabetical order): HD, healthy donor; MBL, monoclonal B-cell lymphocytosis; MBL^{lo}, low-count monoclonal B-cell lymphocytosis; NS, no statistically significant differences found.

Supplementary Table 2. Fluorochrome-conjugated antibody combinations and gating strategy used for flow cytometric identification of the different subsets of leukocytes and B cells circulating in blood of each subject included in this study.

A. LST (EuroFlow® Lymphocyte Screening Tube) BD OneFlow LST™ 8-color tube used for the identification of PB leukocyte populations.

	Fluorochrome-conjugate							
**Antibody reagent**	**V450**	**V500-C**	**FITC**	**PE**	**PerCPCy5.5**	**PECy7**	**APC**	**APC-H7**
**Marker**	CD20<br>CD4	CD45	CD8<br>Anti-λ	CD56<br>Anti-κ	CD5	CD19<br>Anti-TCRγδ	CD3	CD38
**Clone**	L27<br>SK3	2D1	SK1<br>1-155-2	MY31<br>TB28-2	L17F12	SJ25-C1<br>11F2	SK7	HB7
**Source**	BD							

Abbreviations (alphabetical order), APC, allophycocyanine; APC-H7, allophycocyanine-hilite®7; BD, Becton/Dickinson Biosciences; FITC, fluorescein isothiocyanate; PB, peripheral blood; PE, phycoerythrin; PECy7, phycoerythrin-cyanine7; PerCPCy5.5, peridinin chlorophyll protein-cyanine5.5.

B. Immunophenotypic profiles and criteria used for the identification of the major subpopulations of white blood cells identified with the LST antibody combination.

	Cell population								
**FCM marker**	**T-cells**	**B-cells**	**NK-cells**	**Plasma cells**	**Neutrophils**	**Eosinophils**	**Basophils**	**Monocytes**	**Dendritic cells**
**FSC**	low	low	low	int	int / high	int	low	int	low / int
**SSC**	low	low	low	int	high	very high	low	int	low / int
**CD45**	++	++	++	low	+	+	low	+	+
**CD3**	+	-	-	-	-	-	-	-	-
**CD19**	-	+	-	+	-	-	-	-	-
**CD4**	- / +	-	-	-	-	-	-	+	+
**CD8**	- / +	-	-/low	-	-	-	-	-	-
**TCRγδ**	- / +	-	-	-	-	-	-	-	-
**CD20**	-	+	-	- / low	-	-	-	-	-
**CD5**	+/++	- / low	-	-	-	-	-	-	het
**CD56**	het	-	low / +	-	-	-	-	-	-
**CD38**	het	- / low	het	++	-	-	+	low / +	+

Data on the phenotypic profiles of each individual cell population summarized according to reference 3 of Supplementary References. Abbreviations (alphabetical order): FCM, flow cytometry; FSC, forward light scatter; het, heterogeneous expression levels; int, intermediate expression levels; NK, natural killer; SSC, sideward light scatter.

C. EuroFlow® IMM-BIgH 13-color tube used for the identification of different subsets of PB B-cells and plasma cells.

	Fluorochrome-conjugate													
**Ab reagent**	**BV421**	**BV510**	**BV605**	**BV650**	**BV711**	**BV786**	**FITC**	**PerCP-Cy5.5**	**PE**	**PE-CF594**	**PECy7**	**APC**	**AF700**	**APC-H7**
**Marker**	CD27	sm/cy IgM	CD62L	CD24	CD21	CD19	sm/cyIgG3 sm/cyIgG2 sm/cyIgD	sm/cyIgA1 sm/cyIgA2	sm/cyIgG1 sm/cyIgG2	CD20	CD138 CD5	sm/cyIgG4 sm/cyIgA1 sm/cyIgD	CD45	CD38
**Clone**	M-T271	MHM-88	DREG 56	ML5	B-ly4	SJ25C1	SAG3 SAG2 IA6-2	SAA1 SAA2	SAG1 SAG2	2H7	MI15 L17F12	SAA1 SAG4 IA6-2	HI30	HB7
**Source**	BD	Bio-legend	Bio-legend	BD	BD	BD	Cytognos Biolegend	Cytognos	Cytognos	BD	Bio-legend BD	Cytognos BD	BD	BD

Abbreviations (alphabetical order), Ab, antibody; AF, alexa fluor; APC, allophycocyanine; APC-H7, allophycocyanine-hilite®7; BV, brilliant violet; cy, cytoplasmic; FITC, fluorescein isothiocyanate; IMM, immune monitoring; PB, peripheral blood; PE, phycoerythrin; PE-CF594, phycoerythrin-carboxyfluorescein594; PECy7, phycoerythrin-cyanine7; PerCPCy5.5, peridinin chlorophyll protein-cyanine5.5; sm, surface membrane.

D. Immunophenotypic profiles and criteria used for the subsetting of the different B-cell and plasma cell subpopulations identified with IMM-BIgH tube.

	Cell population				
**FCM marker**	**Pre-GC B-cells**		**Memory B-cells**		**Plasma cells**
	**Immature**	**Naïve**	**Unswitched memory**	**Switched memory**	
**FSC**	low	low	low	low	int
**SSC**	low	low	low	low	int
**CD45**	++	++	++	++	low
**CD19**	+	+	+	+	+/low
**CD20**	+	+	+	+	- / +
**CD21**	+	- / +	- / +	- / +	-
**CD24**	++	-	-	-	-
**CD27**	-	-	+	- / +	+
**CD38**	+	-	-	-	++
**CD138**	-	-	-	-	- / +
**CD5**	+	- / +	-	-	-
**IgM/D**	+	+	+	-	- / +

Data on the phenotypic profiles of each individual cell population identified summarized according to reference 6 of Supplementary References.(7) Further subsetting of memory B-cells and plasma cells were performed according to the IgH subclass expressed (IgM, IgD, IgG1, IgG2, IgG3, IgG4, IgA1 and IgA2). Abbreviations (alphabetical order): FCM, flow cytometry; FSC, forward light scatter; GC, germinal center; int, intermediate expression levels; NK, natural killer; SSC, sideward light scatter.

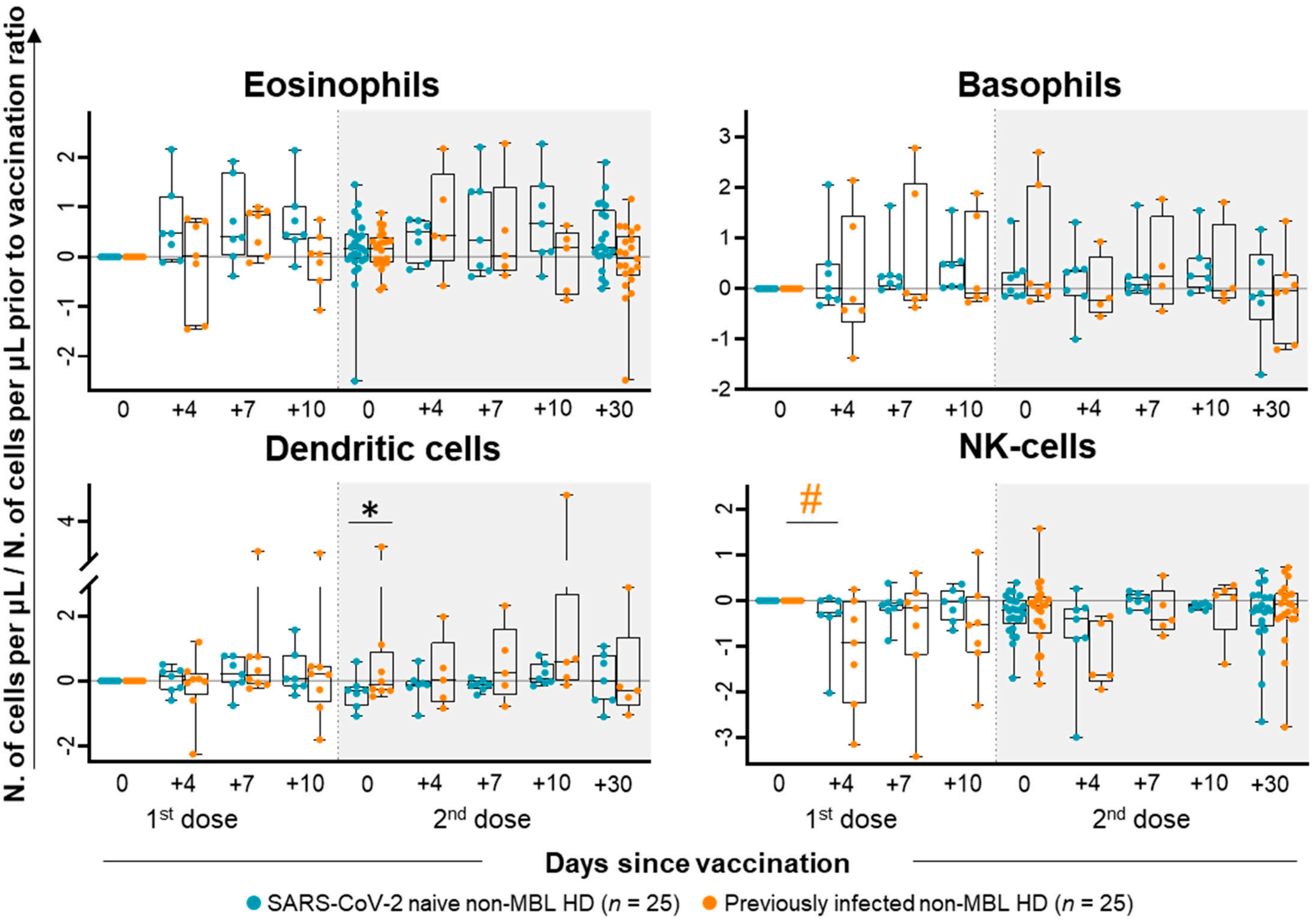

Supplementary Figure 1. Kinetics of eosinophils, basophils, dendritic cells and NK-cells in blood of SARS-CoV-2 naive vs. previously infected non-MBL HD following SARS-CoV-2 vaccination. Data expressed as the ratio (considering 0 as no change vs. pre-vaccination time-point) between the absolute cell count/µL of each cell population and the cell count detected for the same cell population at baseline, prior to vaccination. Subjects without previous contact with SARS-CoV-2 (blue dots) and those who had been previously infected by the virus (orange dots) are grouped according to the number of days from the administration of the vaccine. Notched boxes represent 25th and 75th percentile values (IQR), whereas the line in the middle corresponds to median values, and whiskers represent the maximum and minimum values observed for each group. *Statistically significant differences ($p \leq 0.05$) between SARS-CoV-2 naive vs. previously infected non-MBL HD; #Statistically significant differences ($p \leq 0.05$) between the time-point analyzed and the previous one for each (color-coded) group of individuals. Hashtags (#) depicted in bold refer to statistically significant differences when considering (more stringent) FDR (<5% vs. <10%) for multiple comparisons. Abbreviations: HD, healthy donors; IQR, interquartile range; MBL, monoclonal B-cell lymphocytosis; NK, natural killer.

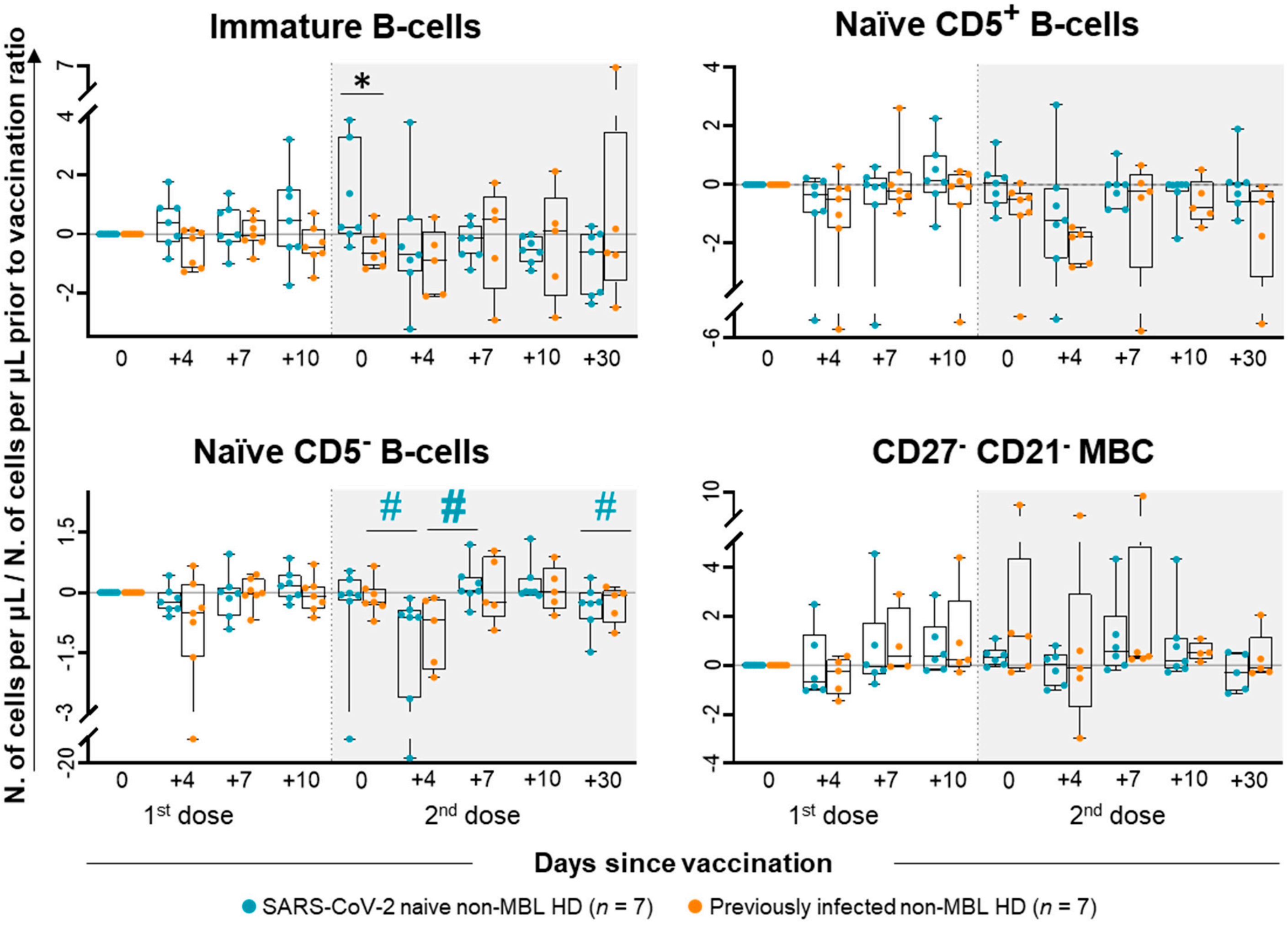

Supplementary Figure 2. Kinetics of pre-germinal center B-cell subsets and CD27⁻CD21⁻ memory B cells in blood of SARS-CoV-2 naïve vs. previously infected non-MBL HD following SARS-CoV-2 vaccination. Data expressed as the ratio (considering 0 as no change vs. pre-vaccination time-point) between absolute cell count/µL of each cell population and the cell count detected for the same cell population at baseline, prior to vaccination. Subjects without previous contact with SARS-CoV-2 (blue dots) and previously infected (orange dots) are grouped according to the number of days from the administration of the vaccine. Notched boxes represent 25th and 75th percentile values (IQR), whereas the line in the middle corresponds to median values, and whiskers represent the maximum and minimum values observed for each group. *Statistically significant differences ($p \leq 0.05$) between SARS-CoV-2 naïve vs. previously infected non-MBL HD; #Statistically significant differences ($p \leq 0.05$) between the time-point analyzed and the previous one for each (color-coded) group of individuals. Hashtags (#) depicted in bold refer to statistically significant differences when considering (more stringent) FDR (<5% vs. <10%) for multiple comparisons. Abbreviations: HD, healthy donors; IQR, interquartile range; MBL, monoclonal B-cell lymphocytosis.

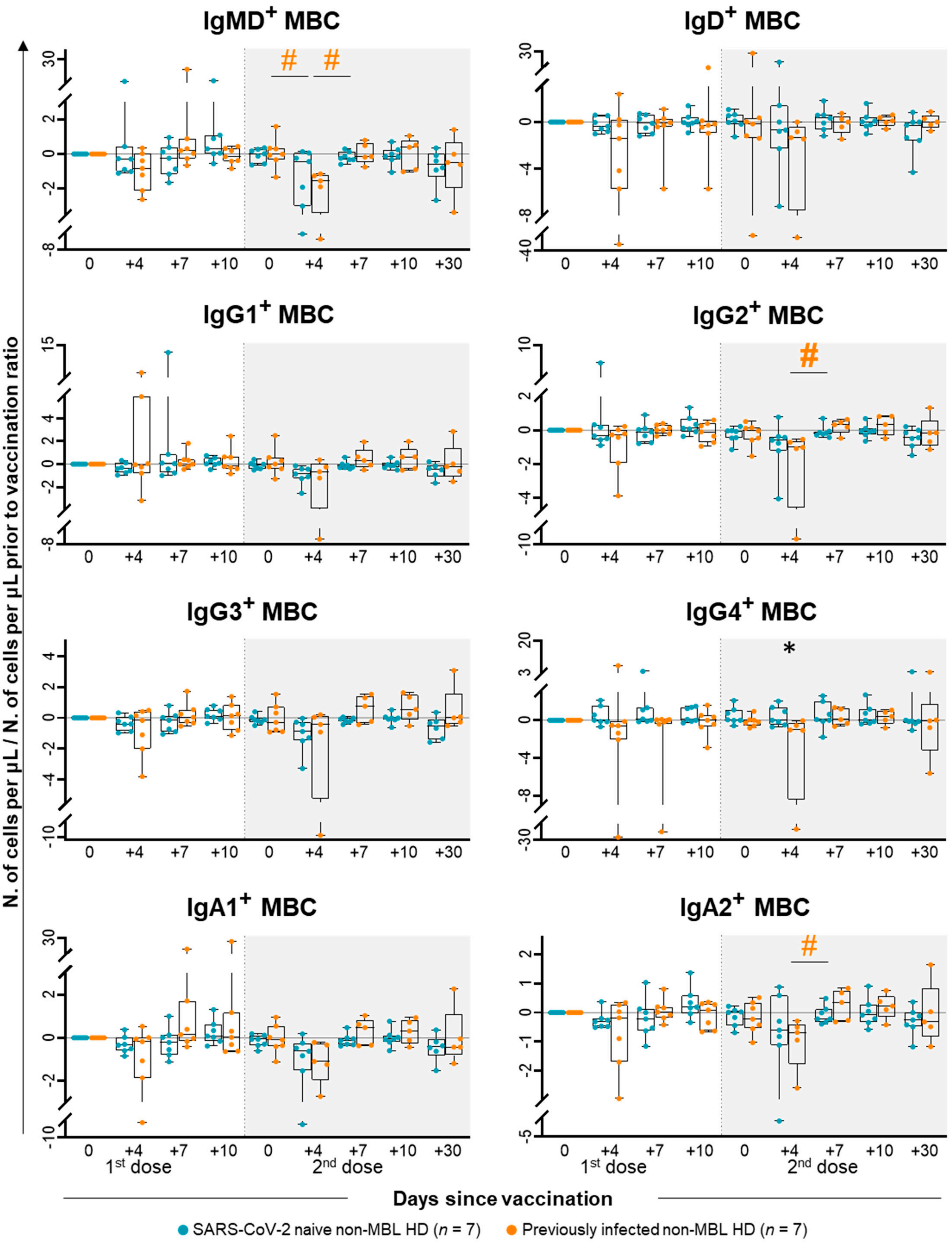

IgMD+ MBC
IgD+ MBC
IgG1+ MBC
IgG2+ MBC
IgG3+ MBC
IgG4+ MBC
IgA1+ MBC
IgA2+ MBC
N. of cells per µL / N. of cells per µL prior to vaccination ratio
0 +4 +7 +10 0 +4 +7 +10 +30
1st dose
2nd dose
Days since vaccination
SARS-CoV-2 naive non-MBL HD (n = 7)
Previously infected non-MBL HD (n = 7)

Supplementary Figure 3. Kinetics of memory B-cell subsets in blood grouped by their IgH isotype and subclass expression profile in blood of SARS-CoV-2 naïve vs. previously infected non-MBL HD following SARS-CoV-2 vaccination. Data expressed as the ratio (considering 0 as no change vs. pre-vaccination time-point) between absolute cell count/µL of each cell population and the cell count detected for the same cell population at baseline, prior to vaccination. Subjects without previous contact with SARS-CoV-2 (blue dots) and previously infected (orange dots) are grouped according to the number of days from the administration of the vaccine. Notched boxes represent 25th and 75th percentile values (IQR), whereas the line in the middle corresponds to median values, and whiskers represent the maximum and minimum values observed for each group. *Statistically significant differences ($p \leq 0.05$) between SARS-CoV-2 naïve vs. previously infected non-MBL HD; #Statistically significant differences ($p \leq 0.05$) between the time-point analyzed and the previous one for each (color-coded) group of individuals. Hashtags (#) depicted in bold refer to statistically significant differences when considering (more stringent) FDR (<5% vs. <10%) for multiple comparisons. Abbreviations: HD, healthy donors; Ig, immunoglobulin; IQR, interquartile range; MBC, memory B-cells; MBL, monoclonal B-cell lymphocytosis.

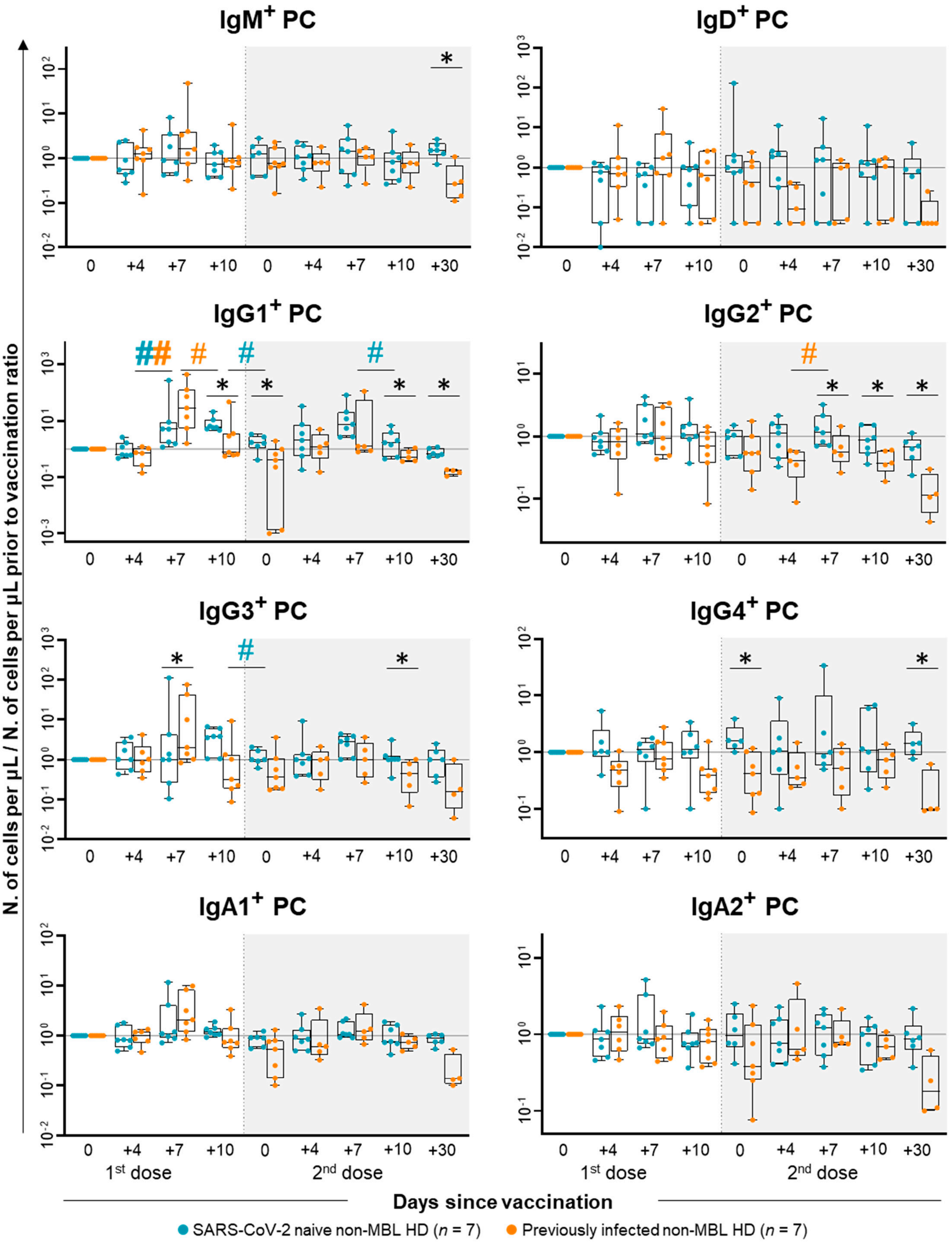
IgM+ PC
IgD+ PC
IgG1+ PC
IgG2+ PC
IgG3+ PC
IgG4+ PC
IgA1+ PC
IgA2+ PC
N. of cells per µL / N. of cells per µL prior to vaccination ratio
0
+4
+7
+10
+30
1st dose
2nd dose
Days since vaccination
SARS-CoV-2 naive non-MBL HD (n = 7)
Previously infected non-MBL HD (n = 7)

Supplementary Figure 4. Kinetics of plasma cell subsets grouped by their IgH isotype and subclass expression profile in blood of SARS-CoV-2 naïve vs. previously infected non-MBL HD following SARS-CoV-2 vaccination. Data expressed as the ratio (considering 0 as no change vs. pre-vaccination time-point) between absolute cell count/µL of each cell population and the cell count detected for the same cell population at baseline, prior to vaccination, using a logarithmic scale. Subjects without previous contact with SARS-CoV-2 (blue dots) and previously infected (orange dots) are grouped according to the number of days from the administration of the vaccine. Notched boxes represent 25th and 75th percentile values (IQR), whereas the line in the middle corresponds to median values, and whiskers represent the maximum and minimum values observed for each group. *Statistically significant differences ($p \leq 0.05$) between SARS-CoV-2 naïve vs. previously infected non-MBL HD; #Statistically significant differences ($p \leq 0.05$) between the time-point analyzed and the previous one for each (color-coded) group of individuals. Hashtags (#) depicted in bold refer to statistically significant differences when considering (more stringent) FDR (<5% vs. <10%) for multiple comparisons. Abbreviations: HD, healthy donors; Ig, immunoglobulin; IQR, interquartile range; MBL, monoclonal B-cell lymphocytosis; PC, plasma cell.

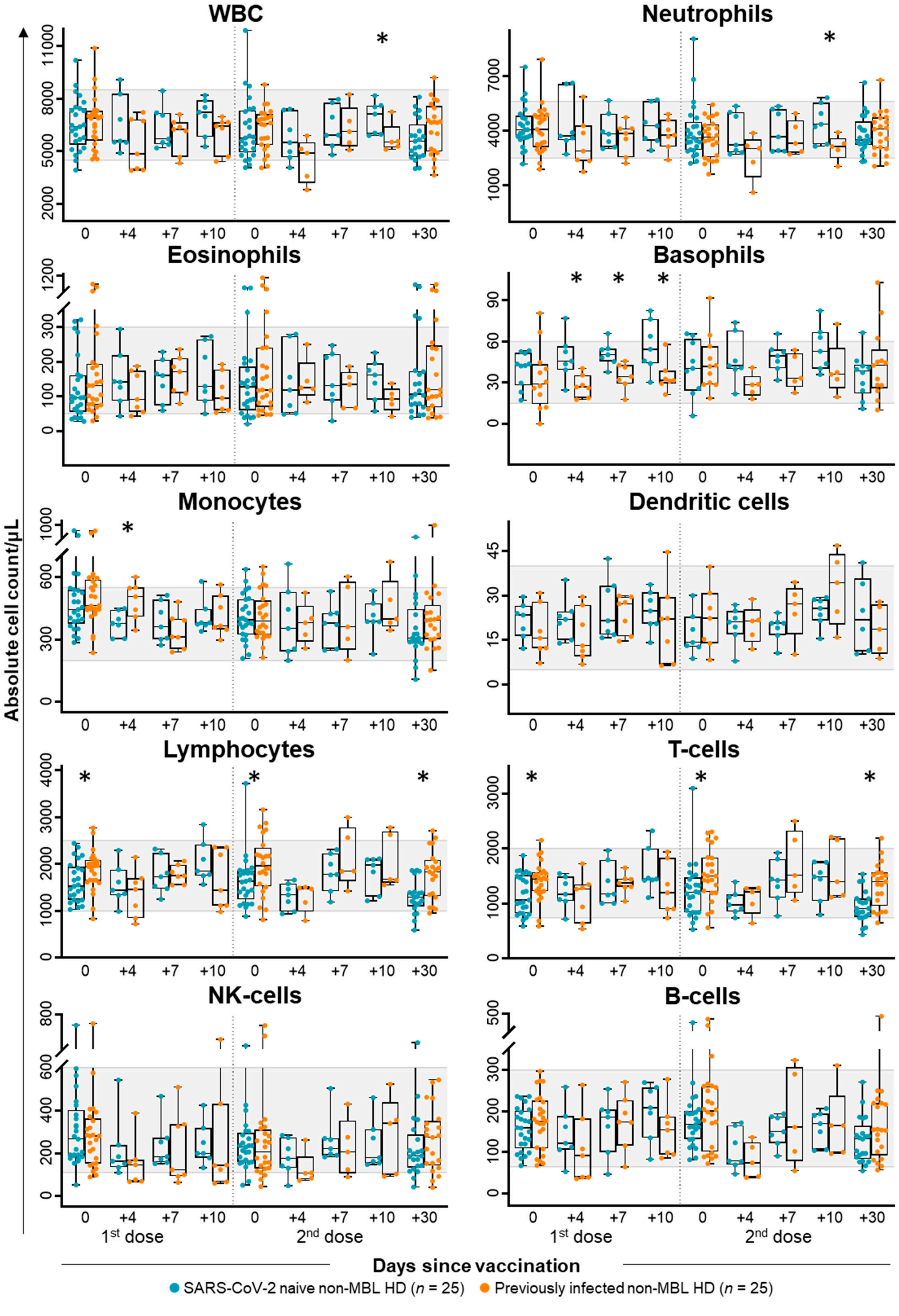

WBC
Neutrophils
Eosinophils
Basophils
Monocytes
Dendritic cells
Lymphocytes
T-cells
NK-cells
B-cells
Absolute cell count/µL
0
+4
+7
+10
+30
1st dose
2nd dose
Days since vaccination
SARS-CoV-2 naive non-MBL HD (n = 25)
Previously infected non-MBL HD (n = 25)

Supplementary Figure 5. Major leukocyte subset kinetics in blood of SARS-CoV-2 naïve vs. previously infected non-MBL HD following SARS-CoV-2 vaccination. Data expressed as absolute cell counts/μL for each individual cell population analyzed. Subjects without previous contact with SARS-CoV-2 (blue dots) and previously infected (orange dots) are grouped according to the number of days from the administration of the vaccine. Notched boxes represent 25th and 75th percentile values (IQR), and whiskers represent the maximum and minimum values observed for each group. Gray horizontal bands represent the 10th-90th percentile values (normality range) in blood of pre-pandemic non-MBL age-matched HD. *Statistically significant differences ($p \leq 0.05$) between SARS-CoV-2 naïve vs. previously infected non-MBL HD. Abbreviations: HD, healthy donors; IQR, interquartile range; MBL, monoclonal B-cell lymphocytosis; NK, natural killer; WBC, white blood cells.

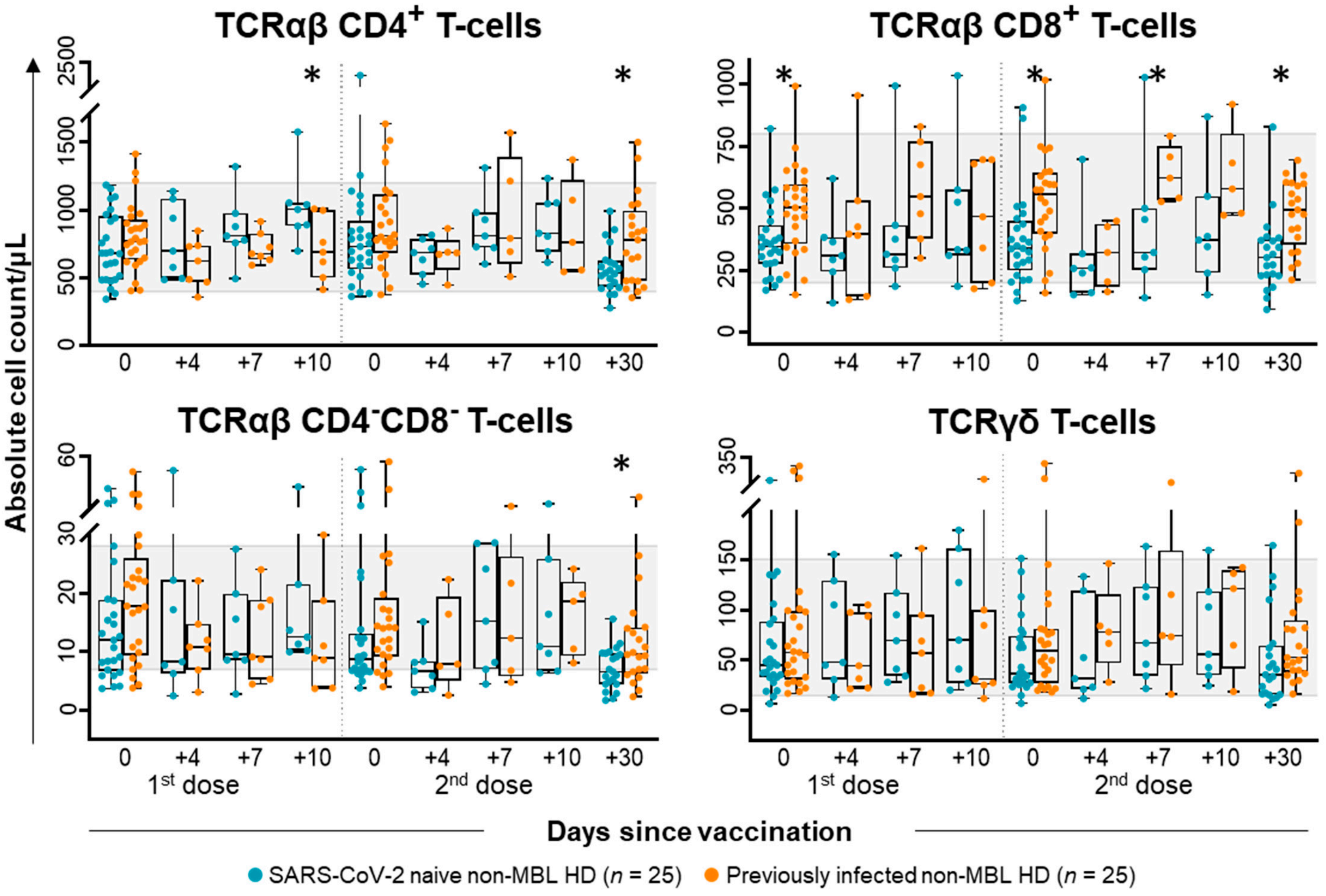

Supplementary Figure 6. Kinetics of T-cell subsets in blood of SARS-CoV-2 naïve vs. previously infected non-MBL HD following SARS-CoV-2 vaccination. Data expressed as absolute cell counts/µL for each individual cell population analyzed. Subjects without previous contact with SARS-CoV-2 (blue dots) and previously infected (orange dots) are grouped according to the number of days from the administration of the vaccine. Notched boxes represent 25th and 75th percentile values (IQR), and whiskers represent the maximum and minimum values observed for each group. Gray horizontal bands represent the 10th-90th percentile values (normality range) in blood of pre-pandemic non-MBL age-matched HD. *Statistically significant differences ($p \leq 0.05$) between SARS-CoV-2 naïve vs. previously infected non-MBL HD. Abbreviations: HD, healthy donors; IQR, interquartile range; MBL, monoclonal B-cell lymphocytosis; TCR, T-cell receptor.

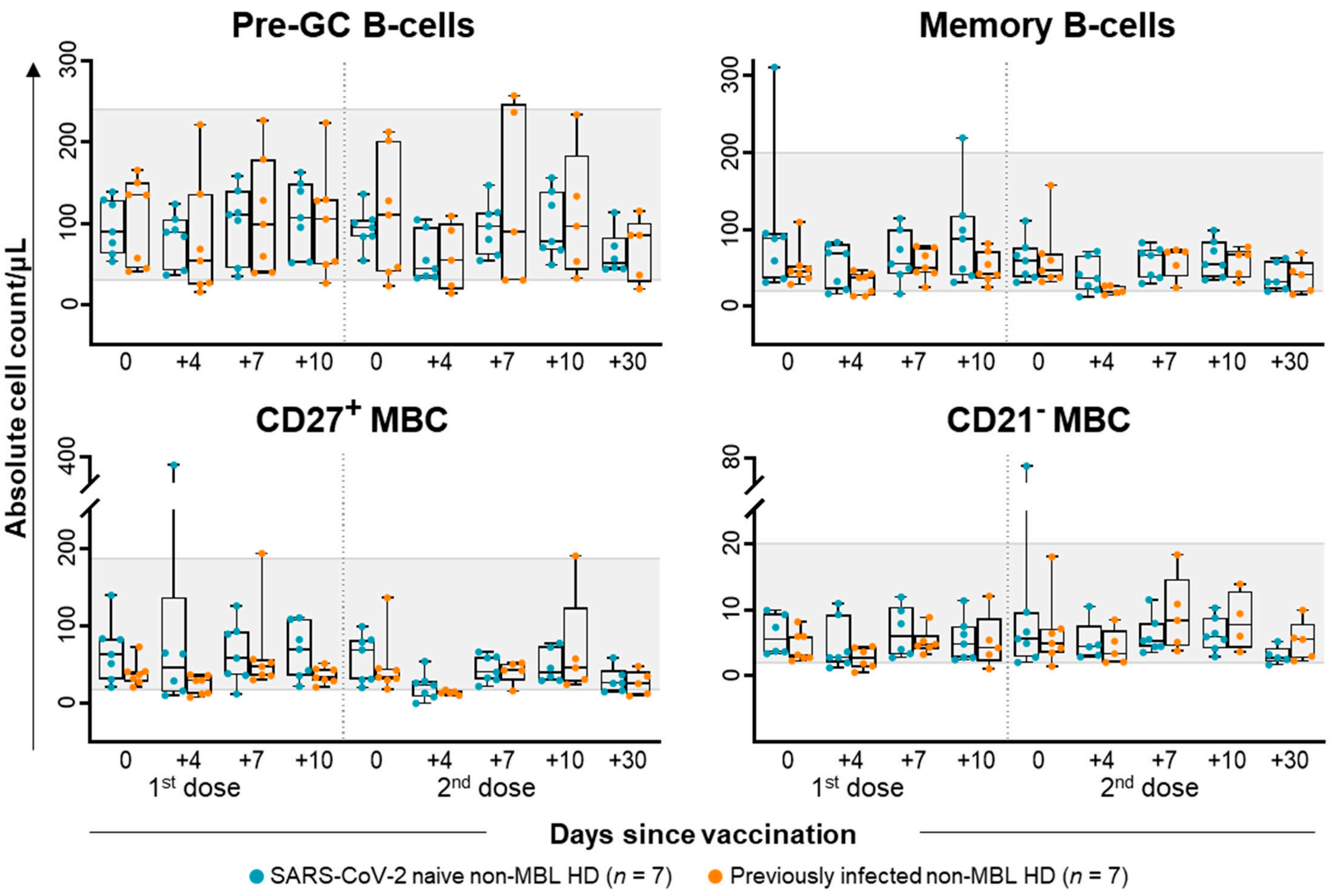

Supplementary Figure 7. Kinetics of B-cell subsets in blood of SARS-CoV-2 naïve vs. previously infected non-MBL HD following SARS-CoV-2 vaccination. Data expressed as absolute cell counts/µL for each individual cell population analyzed. Subjects without previous contact with SARS-CoV-2 (blue dots) and previously infected (orange dots) are grouped according to the number of days from the administration of the vaccine. Notched boxes represent 25th and 75th percentile values (IQR), and whiskers represent the maximum and minimum values observed for each group. Gray horizontal bands represent the 10th-90th percentile values (normality range) in blood of pre-pandemic non-MBL age-matched HD. *Statistically significant differences ($p \leq 0.05$) between SARS-CoV-2 naïve vs. previously infected non-MBL HD. Abbreviations: GC, germinal center; HD, healthy donors; IQR, interquartile range; MBL, monoclonal B-cell lymphocytosis; MBC, memory B-cells.

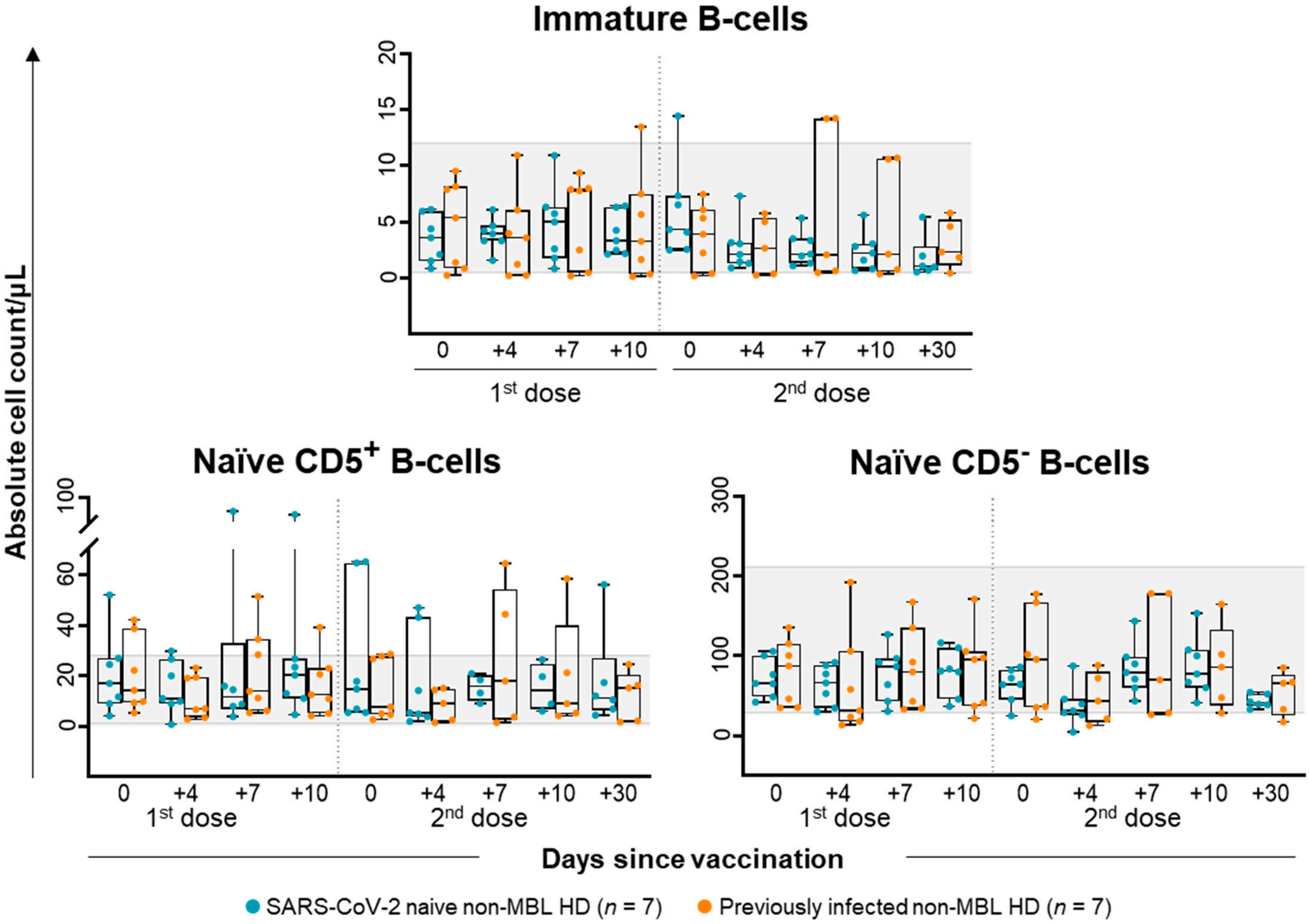

Supplementary Figure 8. Pre-germinal center B-cell subset kinetics in blood of SARS-CoV-2 naïve vs. previously infected non-MBL HD following SARS-CoV-2 vaccination. Data expressed as absolute cell counts/µL for each individual cell population analyzed. Subjects without previous contact with SARS-CoV-2 (blue dots) and previously infected (orange dots) are grouped according to the number of days from the administration of the vaccine. Notched boxes represent 25th and 75th percentile values (IQR), and whiskers represent the maximum and minimum values observed for each group. Gray horizontal bands represent the 10th-90th percentile values (normality range) in blood of pre-pandemic non-MBL age-matched HD. *Statistically significant differences ($p \leq 0.05$) between SARS-CoV-2 naïve vs. previously infected non-MBL HD. Abbreviations: HD, healthy donors; IQR, interquartile range; MBL, monoclonal B-cell lymphocytosis.

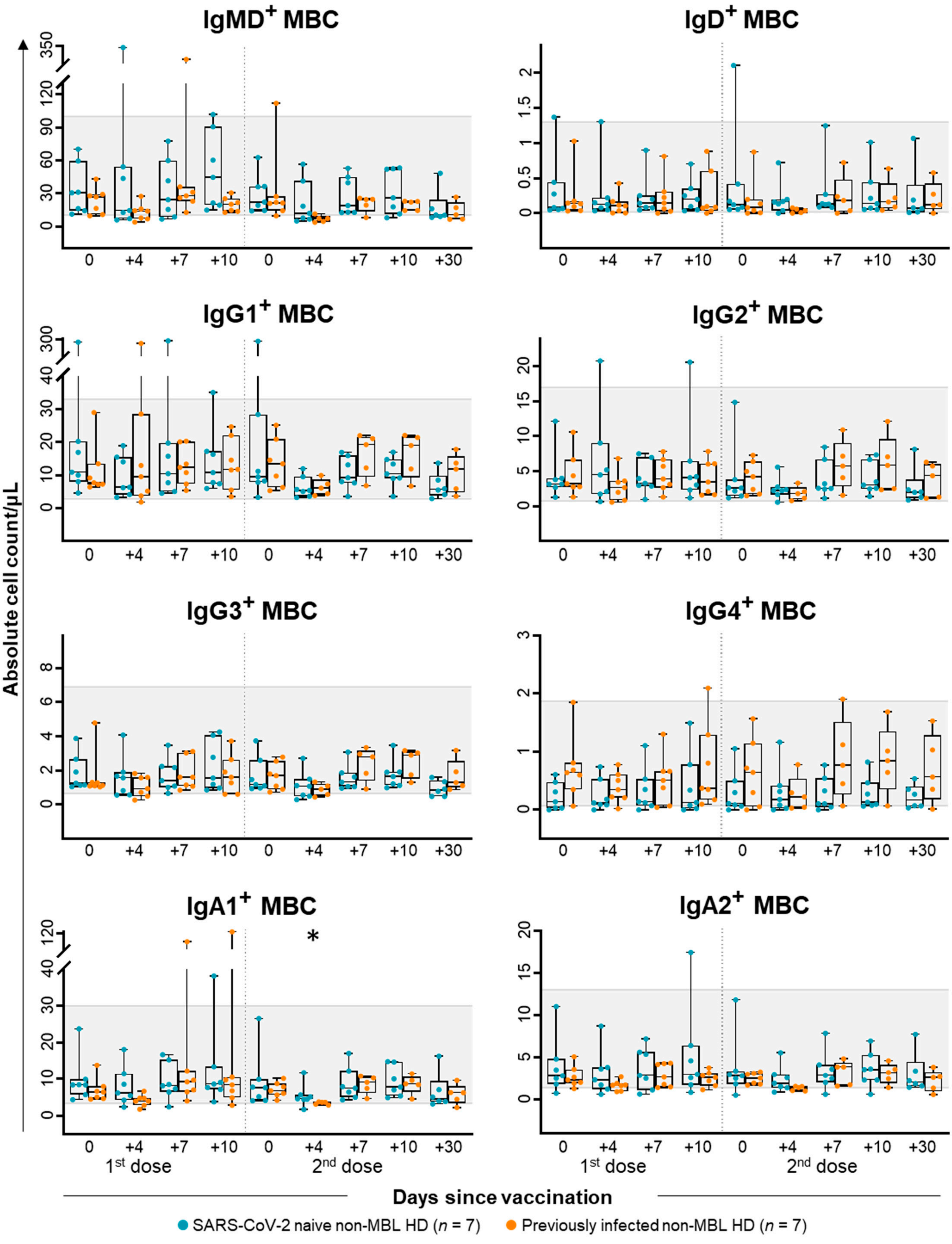
IgMD+ MBC
IgD+ MBC
IgG1+ MBC
IgG2+ MBC
IgG3+ MBC
IgG4+ MBC
IgA1+ MBC
IgA2+ MBC
Absolute cell count/µL
0 +4 +7 +10 0 +4 +7 +10 +30
1st dose
2nd dose
Days since vaccination
SARS-CoV-2 naive non-MBL HD (n = 7)
Previously infected non-MBL HD (n = 7)

Supplementary Figure 9. Kinetics of memory B-cell subsets grouped by their IgH isotype and subclass expression profile in blood of SARS-CoV-2 naïve vs. previously infected non-MBL HD following SARS-CoV-2 vaccination. Data expressed as absolute cell counts/µL for each individual cell population analyzed. Subjects without previous contact with SARS-CoV-2 (blue dots) and previously infected (orange dots) are grouped according to the number of days from the administration of the vaccine. Notched boxes represent 25th and 75th percentile values (IQR), and whiskers represent the maximum and minimum values observed for each group. Gray horizontal bands represent the 10th-90th percentile values (normality range) in blood of pre-pandemic non-MBL age-matched HD. *Statistically significant differences ($p \leq 0.05$) between SARS-CoV-2 naïve vs. previously infected non-MBL HD. Abbreviations: HD, healthy donors; Ig, immunoglobulin; IQR, interquartile range; MBC, memory B-cell; MBL, monoclonal B-cell lymphocytosis.

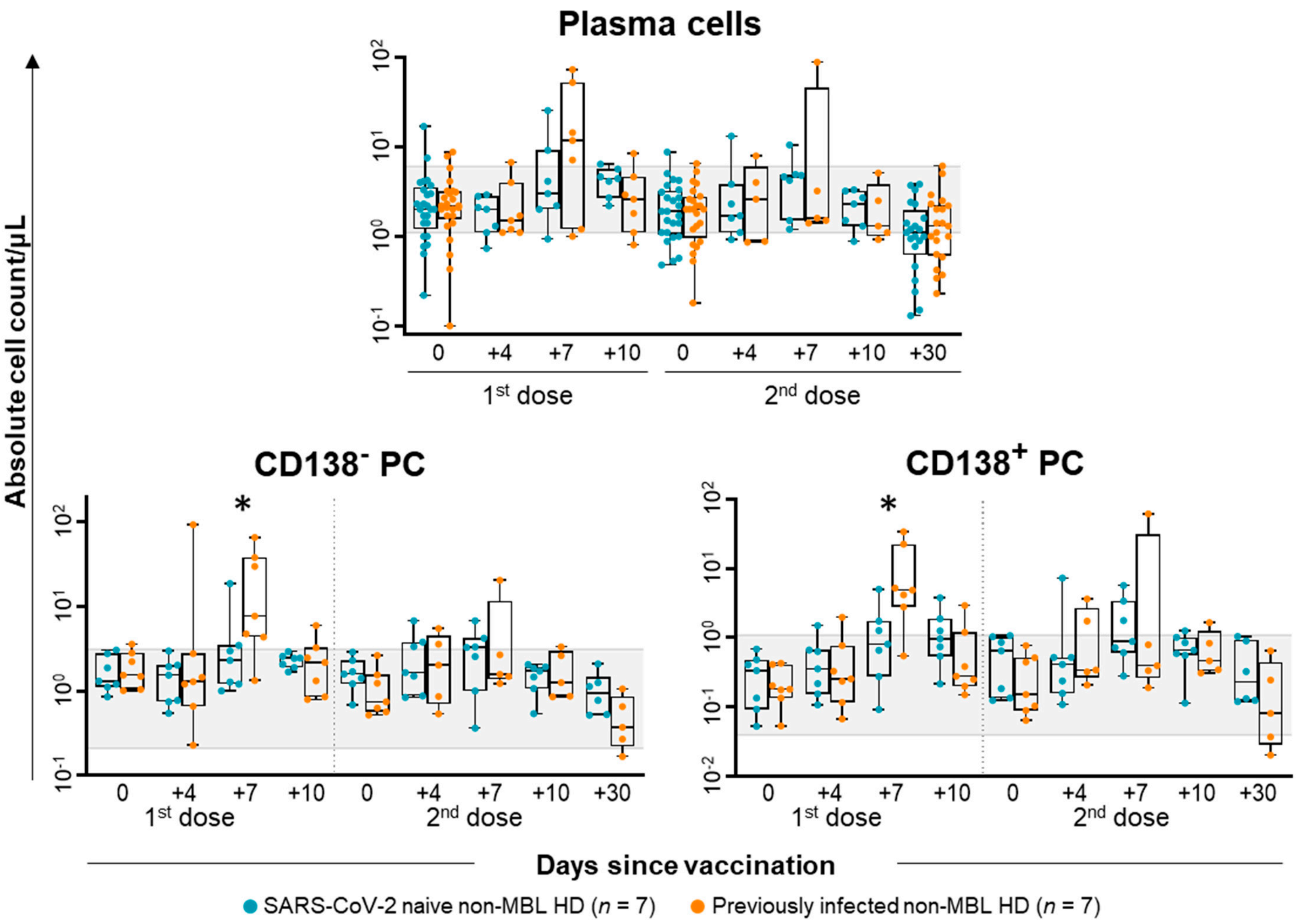

Supplementary Figure 10. Kinetics of plasma cell subsets grouped by their maturation-associated subsets in blood of SARS-CoV-2 naïve vs. previously infected non-MBL HD following SARS-CoV-2 vaccination. Data expressed as absolute cell counts/µL for each individual cell population analyzed, using a logarithmic scale. Subjects without previous contact with SARS-CoV-2 (blue dots) and previously infected (orange dots) are grouped according to the number of days from the administration of the vaccine. Notched boxes represent 25th and 75th percentile values (IQR), and whiskers represent the maximum and minimum values observed for each group. Gray horizontal bands represent the 10th-90th percentile values (normality range) in blood of pre-pandemic non-MBL age-matched HD. *Statistically significant differences ($p \leq 0.05$) between SARS-CoV-2 naïve vs. previously infected non-MBL HD. Abbreviations: HD, healthy donors; IQR, interquartile range; MBL, monoclonal B-cell lymphocytosis; PC, plasma cells.

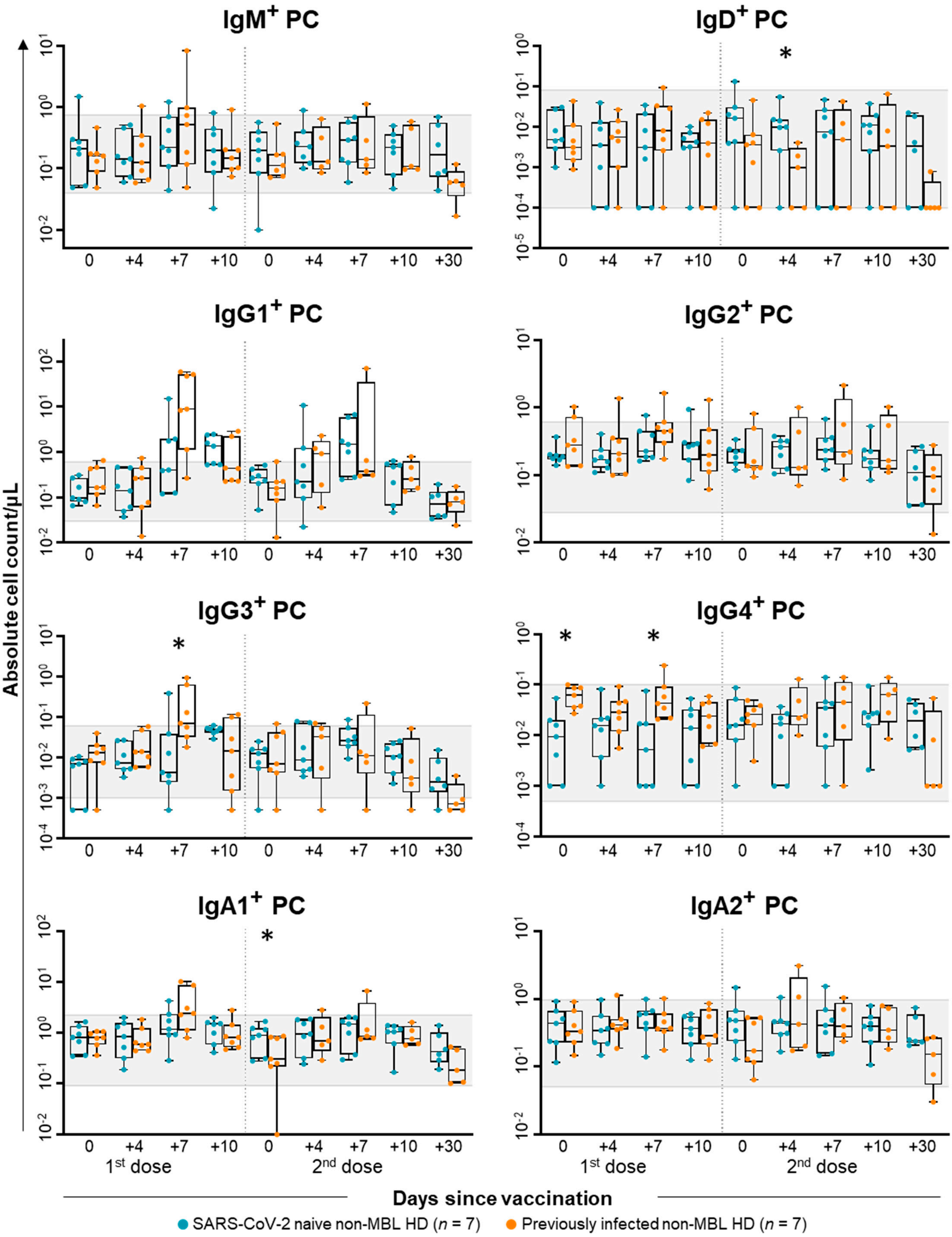
IgM+ PC
IgD+ PC
IgG1+ PC
IgG2+ PC
IgG3+ PC
IgG4+ PC
IgA1+ PC
IgA2+ PC
Absolute cell count/µL
0 +4 +7 +10 0 +4 +7 +10 +30
1st dose
2nd dose
Days since vaccination
SARS-CoV-2 naive non-MBL HD (n = 7)
Previously infected non-MBL HD (n = 7)

Supplementary Figure 11. Kinetics of plasma cell subsets defined by their IgH isotype and subclass expression profile in blood of SARS-CoV-2 naïve vs. previously infected non-MBL HD following SARS-CoV-2 vaccination. Data expressed as absolute cell counts/µL for each individual cell population analyzed, using a logarithmic scale. Subjects without previous contact with SARS-CoV-2 (blue dots) and previously infected (orange dots) are grouped according to the number of days from the administration of the vaccine. Notched boxes represent 25th and 75th percentile values (IQR), and whiskers represent the maximum and minimum values observed for each group. Gray horizontal bands represent the 10th-90th percentile values (normality range) in blood of pre-pandemic non-MBL age-matched HD. *Statistically significant differences ($p \leq 0.05$) between SARS-CoV-2 naïve vs. previously infected non-MBL HD. Abbreviations: HD, healthy donors; Ig, immunoglobulin; IQR, interquartile range; MBL, monoclonal B-cell lymphocytosis; PC, plasma cells.

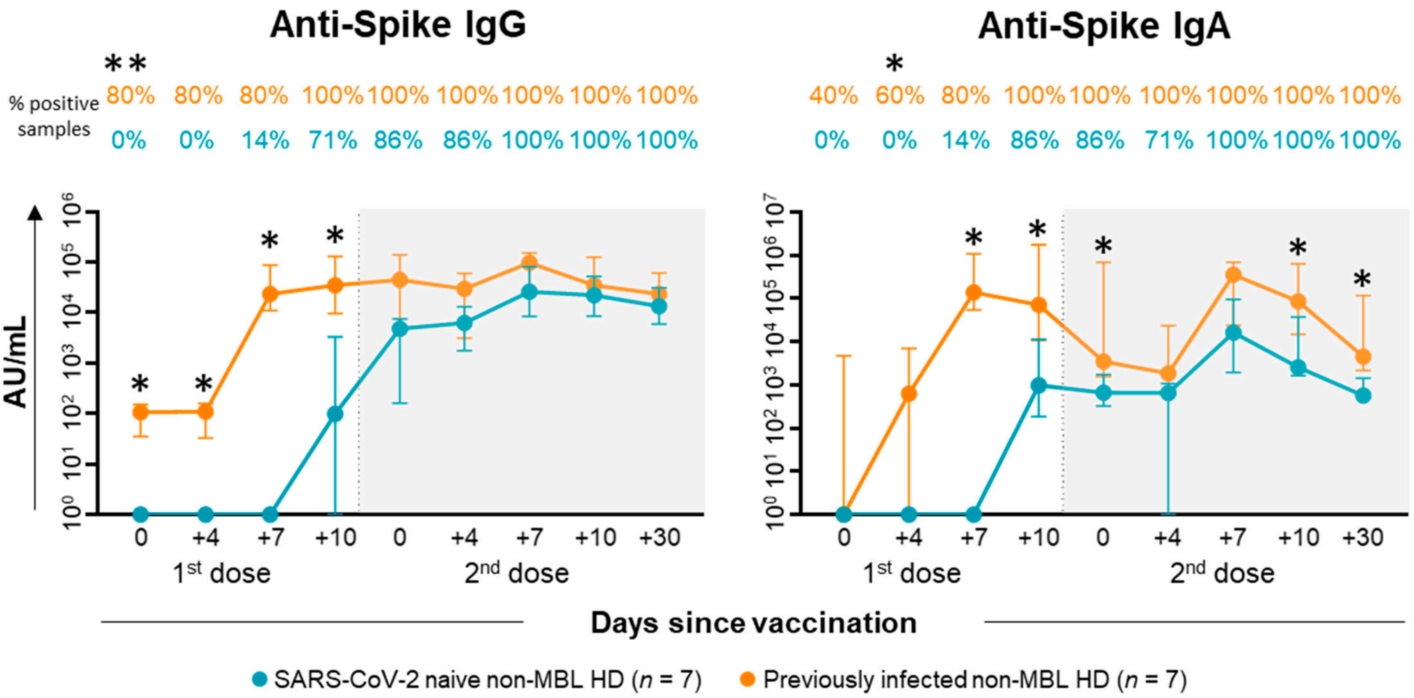

Supplementary Figure 12. Kinetics of SARS-CoV-2 specific antibody levels in plasma of a subgroup of SARS-CoV-2 naïve vs. previously infected non-MBL HD in whom closer sequential monitoring of anti-Spike SARS-CoV-2 antibodies following vaccination was performed (n=14). Plasma levels (AU/ml) of anti-spike SARS-CoV-2 specific IgG and IgA antibodies in SARS-CoV-2 naive vs. previous COVID-19 non-MBL HD with (paired) data available for all time points analyzed for the closer follow-up, using a logarithmic scale. Subjects without previous contact with SARS-CoV-2 (blue dots) and previously infected (orange dots) are grouped according to the number of days from the administration of the vaccine. Whiskers represent 25th and 75th percentile values (IQR), whereas the point in the middle corresponds to median values. Percentages in the figure panels indicate the proportion of samples with detectable antibodies in plasma. Statistically significant differences between SARS-CoV-2 naive vs. previously infected non-MBL HD are represented with asterisks (p-value: * p≤0.05, ** p≤0.01, *** p≤0.001); #Statistically significant differences (p≤0.05) between the time-point analyzed and the previous one for each (color-coded) group of individuals. Abbreviations: AU, arbitrary units; HD, healthy donors; Ig, immunoglobulin; IQR, interquartile range; MBL, monoclonal B-cell lymphocytosis.

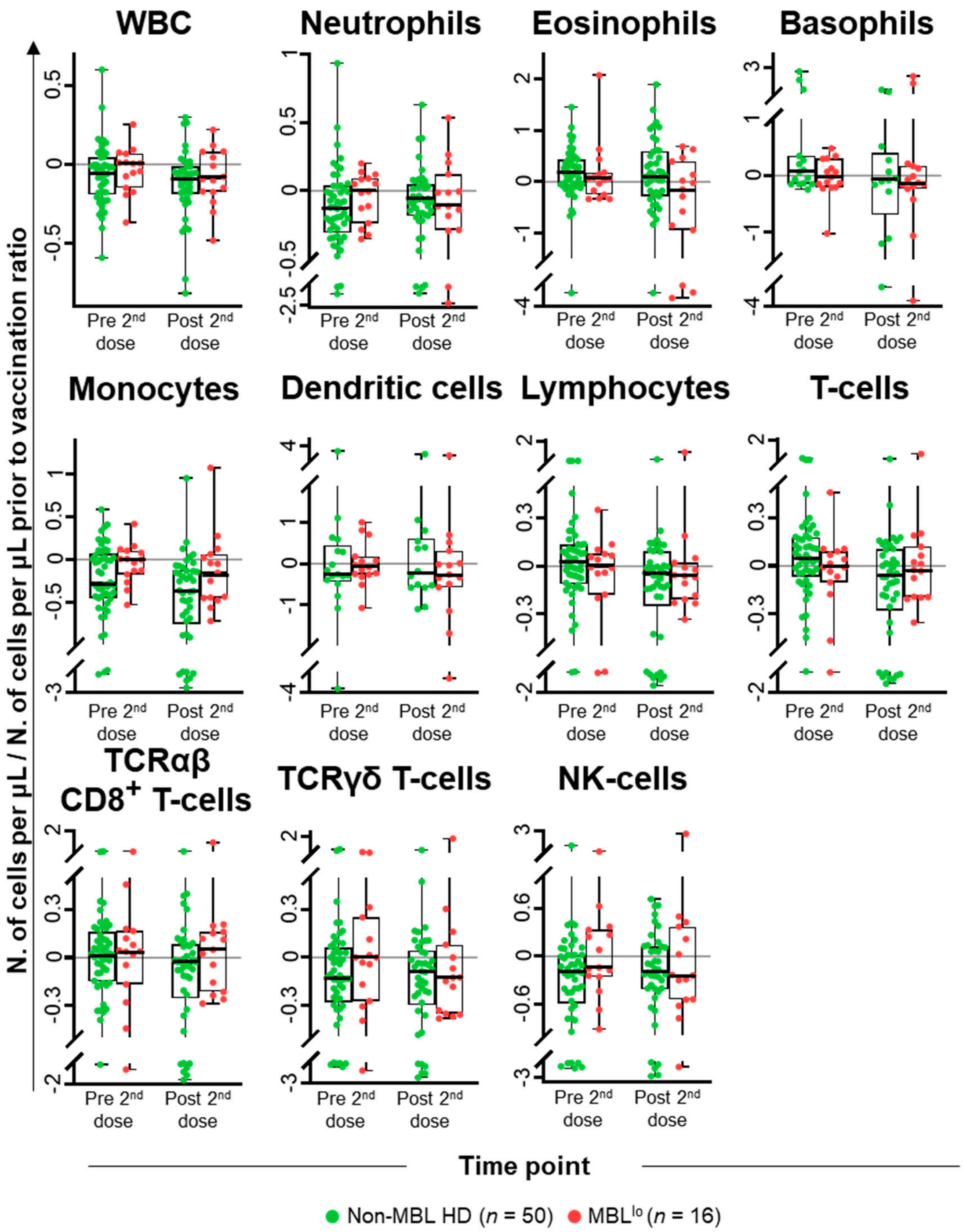

Supplementary Figure 13. Kinetics of the major populations of leukocytes in blood of MBL^{lo} vs. non-MBL HD after SARS-CoV-2 vaccination (independently of the SARS-CoV-2 infection status before vaccination). Data expressed as the ratio (considering 0 as no change vs. pre-vaccination time-point) between the absolute cell count/µL of each cell population and the cell count detected for the same cell population at baseline, prior to vaccination. Subjects without MBL (green dots) and with MBL^{lo} (red dots) are grouped according to the time from the administration of the vaccine. Notched boxes represent 25th and 75th percentile values (IQR), whereas the line in the middle corresponds to median values, and whiskers represent the maximum and minimum values observed for each group. *Statistically significant differences ($p \leq 0.05$) between MBL^{lo} vs. non-MBL HD. Abbreviations: HD, healthy donors; IQR, interquartile range; MBL, monoclonal B-cell lymphocytosis; TCR, T-cell receptor.

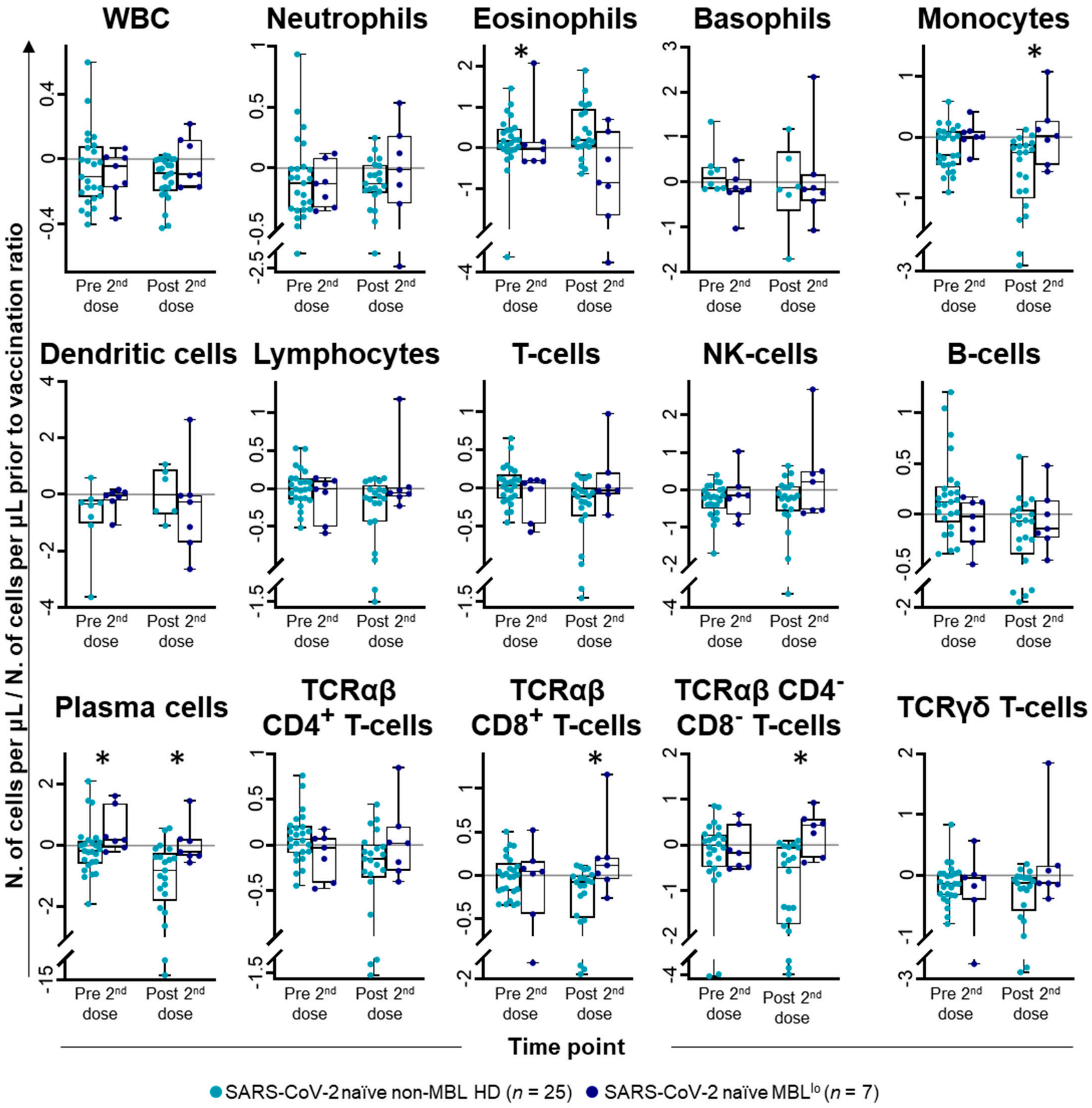

Supplementary Figure 14. Leukocyte subset kinetics in blood of SARS-CoV-2 naïve MBL^{lo} vs. non-MBL HD following SARS-CoV-2 vaccination. Data expressed as the ratio (considering 0 as no change vs. pre-vaccination time-point) between absolute cell count/μL of each cell population and the cell count detected for the same cell population at baseline, prior to vaccination. Subjects without MBL (light blue dots) and with MBL^{lo} (dark blue dots) are grouped according to the time from the vaccine administration. Notched boxes represent 25th and 75th percentile values (IQR), whereas the line in the middle corresponds to median values, and whiskers represent the maximum and minimum values observed for each group. *Statistically significant differences ($p \leq 0.05$) between MBL^{lo} vs. non-MBL HD Abbreviations: HD, healthy donors; IQR, interquartile range; MBL, monoclonal B-cell lymphocytosis; NK, natural killer; TCR, T-cell receptor.

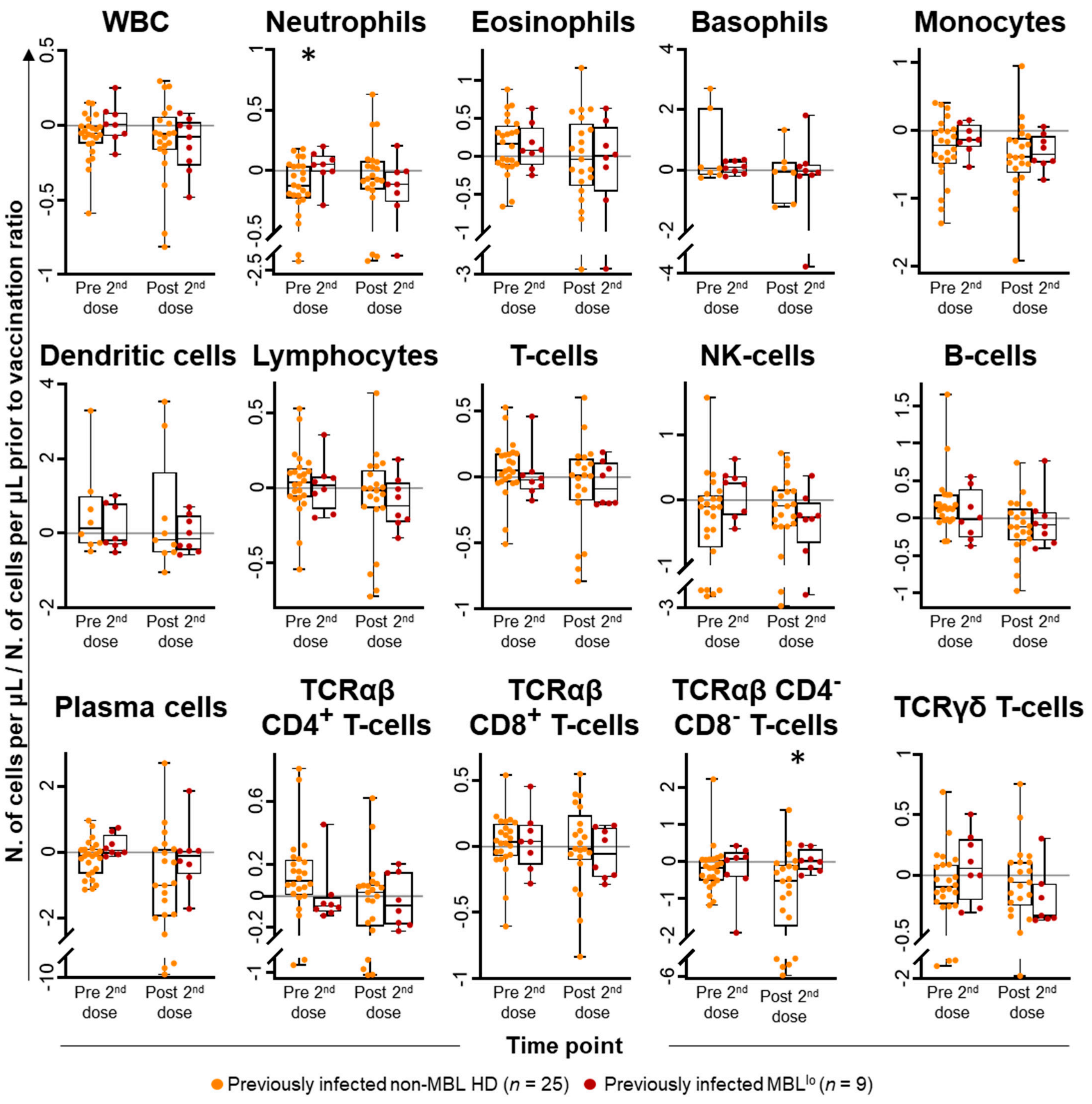

Supplementary Figure 15. Leukocyte subset kinetics in blood of previously infected MBL^{lo} vs. non-MBL HD following SARS-CoV-2 vaccination. Data expressed as the ratio (considering 0 as no change vs. pre-vaccination time-point) between absolute cell count/µL of each cell population and the cell count detected for the same cell population at baseline, prior to vaccination. Subjects without MBL (orange dots) and with MBL^{lo} (red dots) are grouped according to the time from the vaccine administration. Notched boxes represent 25th and 75th percentile values (IQR), whereas the line in the middle corresponds to median values, and whiskers represent the maximum and minimum values observed for each group. *Statistically significant differences ($p \leq 0.05$) between MBL^{lo} vs. non-MBL HD Abbreviations: HD, healthy donors; IQR, interquartile range; MBL, monoclonal B-cell lymphocytosis; NK, natural killer; TCR, T-cell receptor.

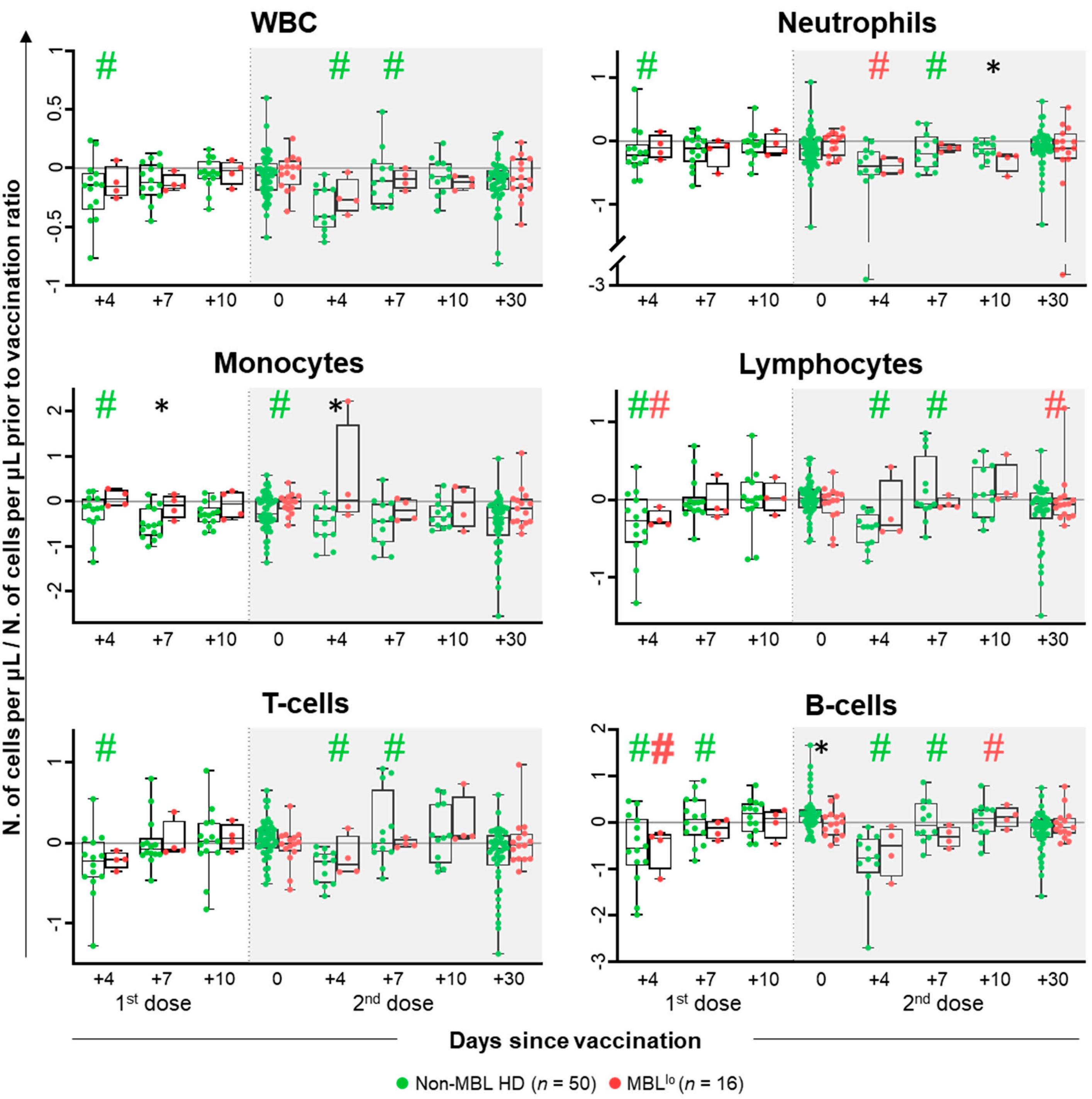

WBC
Neutrophils
Monocytes
Lymphocytes
T-cells
B-cells
N. of cells per µL / N. of cells per µL prior to vaccination ratio
+4 +7 +10 0 +4 +7 +10 +30
1st dose
2nd dose
Days since vaccination
Non-MBL HD (n = 50)
MBLlo (n = 16)

Supplementary Figure 16. Longitudinal kinetics of the major populations of leukocytes in blood of MBL^{lo} vs. non-MBL HD after SARS-CoV-2 vaccination (independently of the SARS-CoV-2 infection status before vaccination). Data expressed as the ratio (considering 0 as no change vs. pre-vaccination time-point) between the absolute cell count/μL of each cell population and the cell count detected for the same cell population at baseline, prior to vaccination. Subjects without MBL (green dots) and with MBL^{lo} (red dots) with data available for all time points analyzed, are grouped according to the time from the administration of the vaccine. Notched boxes represent 25th and 75th percentile values (IQR), whereas the line in the middle corresponds to median values, and whiskers represent the maximum and minimum values observed for each group. *Statistically significant differences ($p \leq 0.05$) between SARS-CoV-2 naive vs. previously infected non-MBL HD; #Statistically significant differences ($p \leq 0.05$) between the time-point analyzed and the previous one for each (color-coded) group of individuals. Hashtags (#) depicted in bold refer to statistically significant differences when considering (more stringent) FDR (<5% vs. <10%) for multiple comparisons. Abbreviations: HD, healthy donors; IQR, interquartile range; MBL, monoclonal B-cell lymphocytosis; WBC, white blood cells.

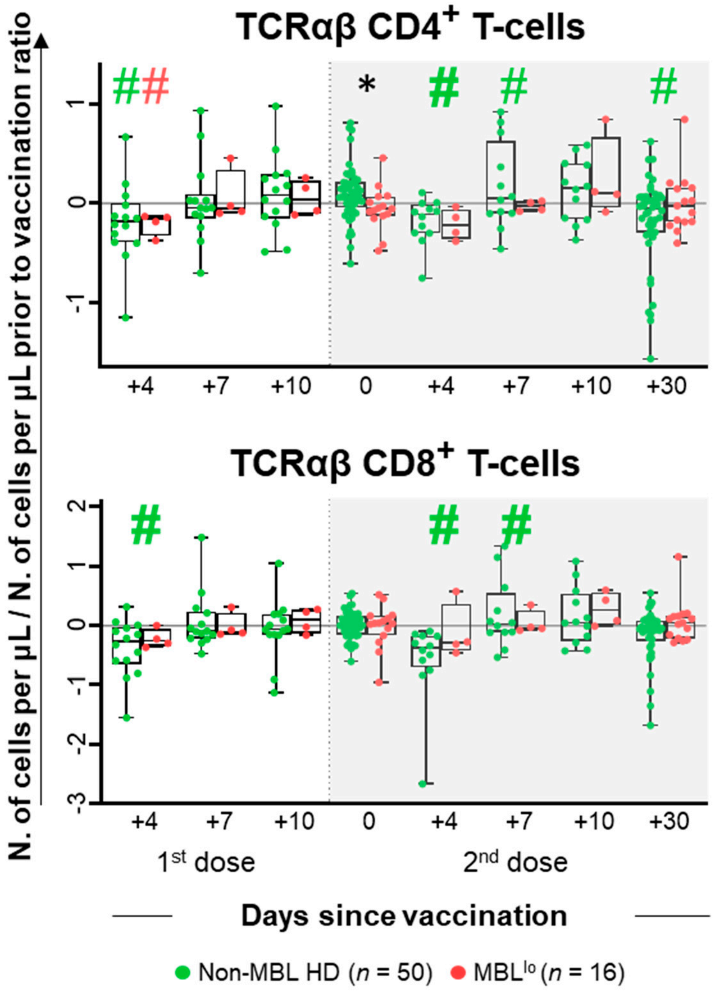

Supplementary Figure 17. Longitudinal kinetics of the major CD4$^+$ and CD8$^+$ T-cell subsets in blood of MBL^{lo} vs. non-MBL HD after SARS-CoV-2 vaccination (independently of the SARS-CoV-2 infection status before vaccination). Data expressed as the ratio (considering 0 as no change vs. pre-vaccination time-point) between the absolute cell count/µL of each cell population and the cell count detected for the same cell population at baseline, prior to vaccination. Subjects without MBL (green dots) and with MBL^{lo} (red dots) with data available for all time points analyzed, are grouped according to the time from the administration of the vaccine. Notched boxes represent 25th and 75th percentile values (IQR), whereas the line in the middle corresponds to median values, and whiskers represent the maximum and minimum values observed for each group. *Statistically significant differences ($p \leq 0.05$) between SARS-CoV-2 naive vs. previously infected non-MBL HD; #Statistically significant differences ($p \leq 0.05$) between the time-point analyzed and the previous one for each (color-coded) group of individuals. Hashtags (#) depicted in bold refer to statistically significant differences when considering (more stringent) FDR (<5% vs. <10%) for multiple comparisons. Abbreviations: HD, healthy donors; IQR, interquartile range; MBL, monoclonal B-cell lymphocytosis; TCR, T-cell receptor.

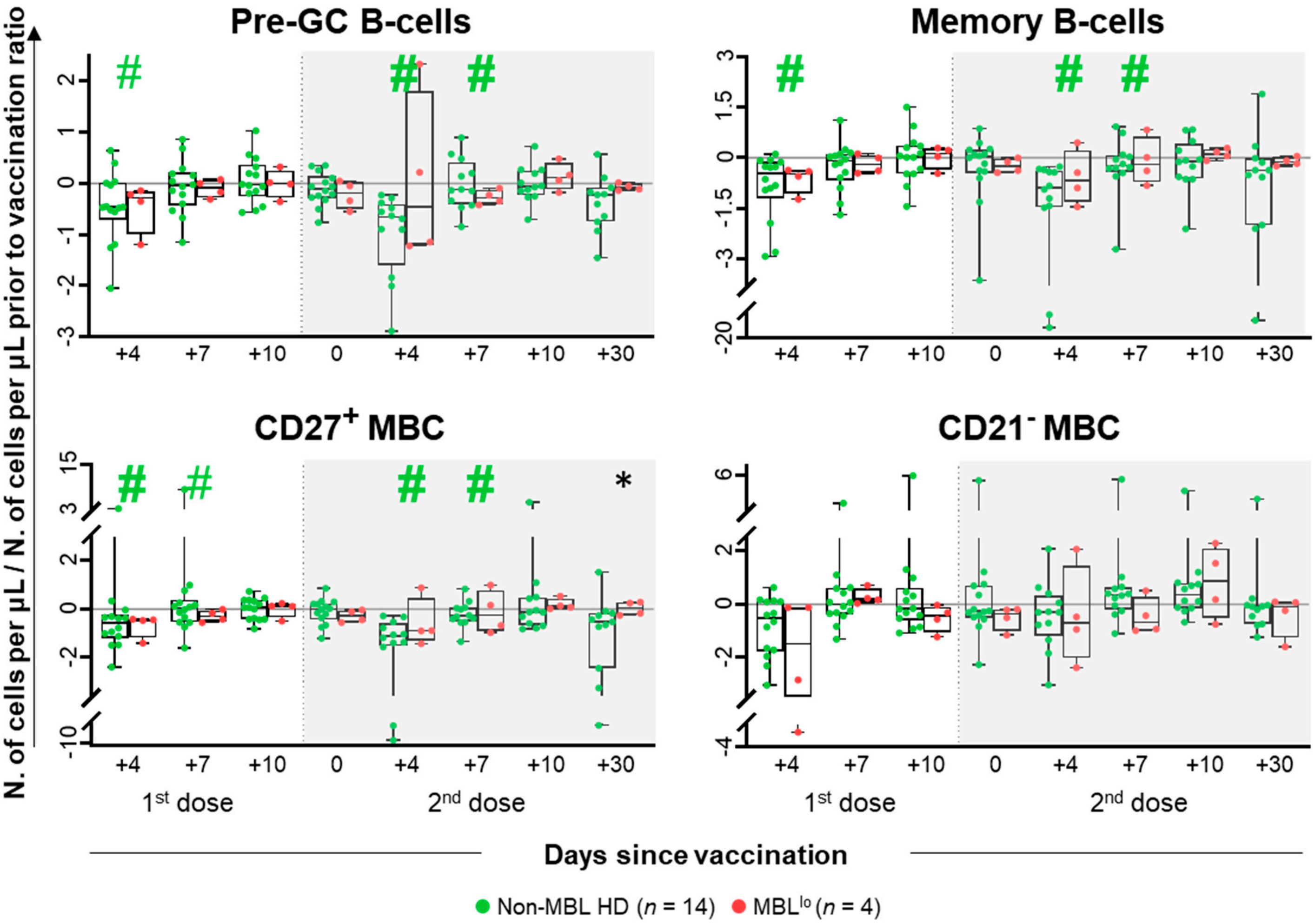

Supplementary Figure 18. Longitudinal kinetics of different B-cell subsets in blood of MBL^{lo} vs. non-MBL HD after SARS-CoV-2 vaccination (independently of the SARS-CoV-2 infection status before vaccination). Data expressed as the ratio (considering 0 as no change vs. pre-vaccination time-point) between the absolute cell count/μL of each cell population and the cell count detected for the same cell population at baseline, prior to vaccination. Subjects without MBL (green dots) and with MBL^{lo} (red dots) with data available for all time points analyzed, are grouped according to the time from the administration of the vaccine. Notched boxes represent 25th and 75th percentile values (IQR), whereas the line in the middle corresponds to median values, and whiskers represent the maximum and minimum values observed for each group. *Statistically significant differences ($p \leq 0.05$) between SARS-CoV-2 naive vs. previously infected non-MBL HD; #Statistically significant differences ($p \leq 0.05$) between the time-point analyzed and the previous one for each (color-coded) group of individuals. Hashtags (#) depicted in bold refer to statistically significant differences when considering (more stringent) FDR (<5% vs. <10%) for multiple comparisons. Abbreviations: GC, germinal center; HD, healthy donors; IQR, interquartile range; MBL, monoclonal B-cell lymphocytosis; MBC, memory B-cells.

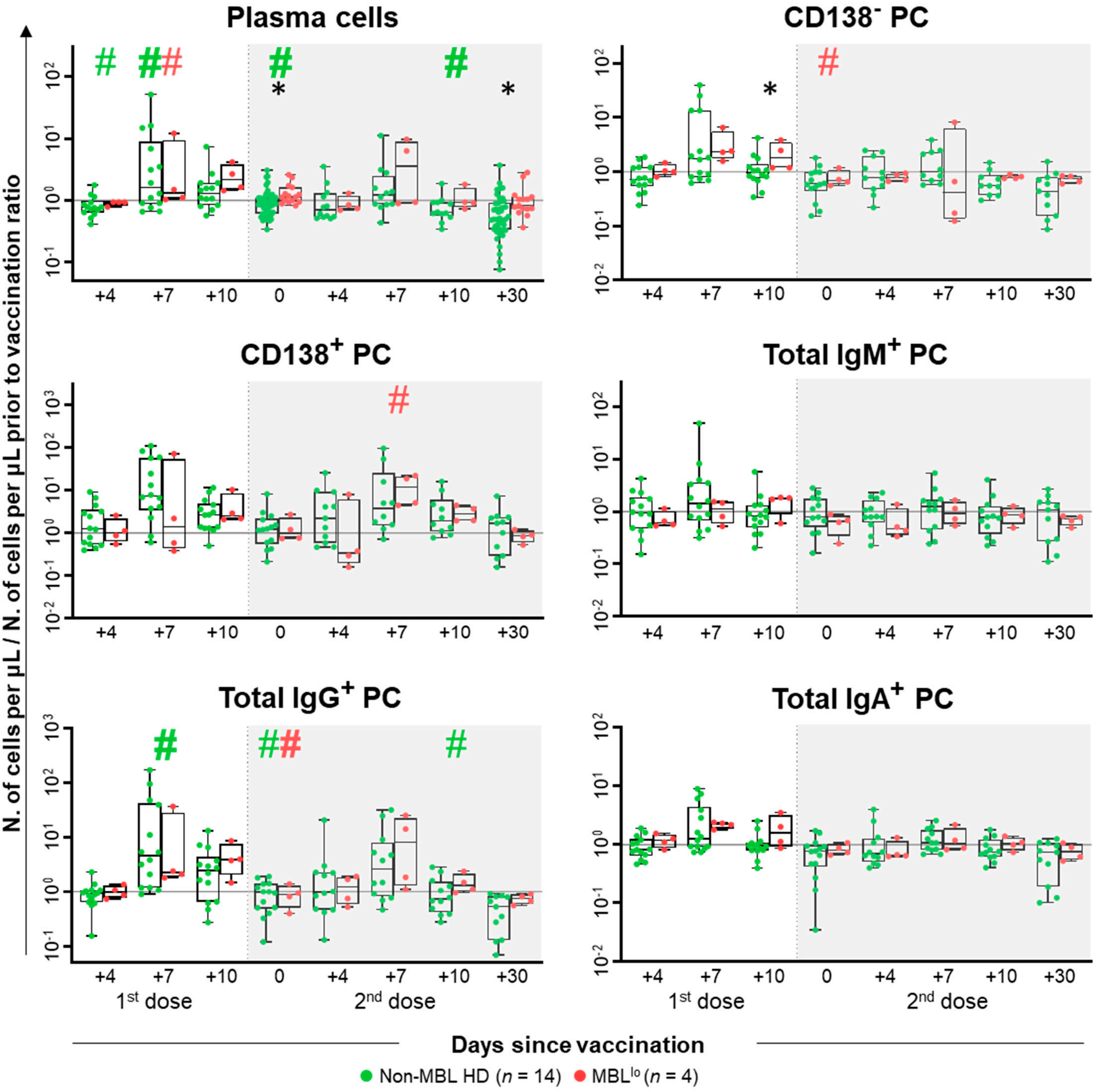

Plasma cells
CD138- PC
CD138+ PC
Total IgM+ PC
Total IgG+ PC
Total IgA+ PC
N. of cells per µL / N. of cells per µL prior to vaccination ratio
+4
+7
+10
0
+30
1st dose
2nd dose
Days since vaccination
Non-MBL HD (n = 14)
MBLlo (n = 4)

Supplementary Figure 19. Longitudinal kinetics of different plasma cell subsets in blood of MBL^{lo} vs. non-MBL HD after SARS-CoV-2 vaccination (independently of the SARS-CoV-2 infection status before vaccination). Data expressed as the ratio (considering 0 as no change vs. pre-vaccination time-point) between the absolute cell count/µL of each cell population and the cell count detected for the same cell population at baseline, prior to vaccination. Subjects without MBL (green dots) and with MBL^{lo} (red dots) with data available for all time points analyzed, are grouped according to the time from the administration of the vaccine. Notched boxes represent 25th and 75th percentile values (IQR), whereas the line in the middle corresponds to median values, and whiskers represent the maximum and minimum values observed for each group. *Statistically significant differences ($p \leq 0.05$) between SARS-CoV-2 naive vs. previously infected non-MBL HD; #Statistically significant differences ($p \leq 0.05$) between the time-point analyzed and the previous one for each (color-coded) group of individuals. Hashtags (#) depicted in bold refer to statistically significant differences when considering a (more stringent) FDR <5%. Abbreviations: HD, healthy donors; Ig, immunoglobulin; IQR, interquartile range; MBL, monoclonal B-cell lymphocytosis; PC, plasma cells.

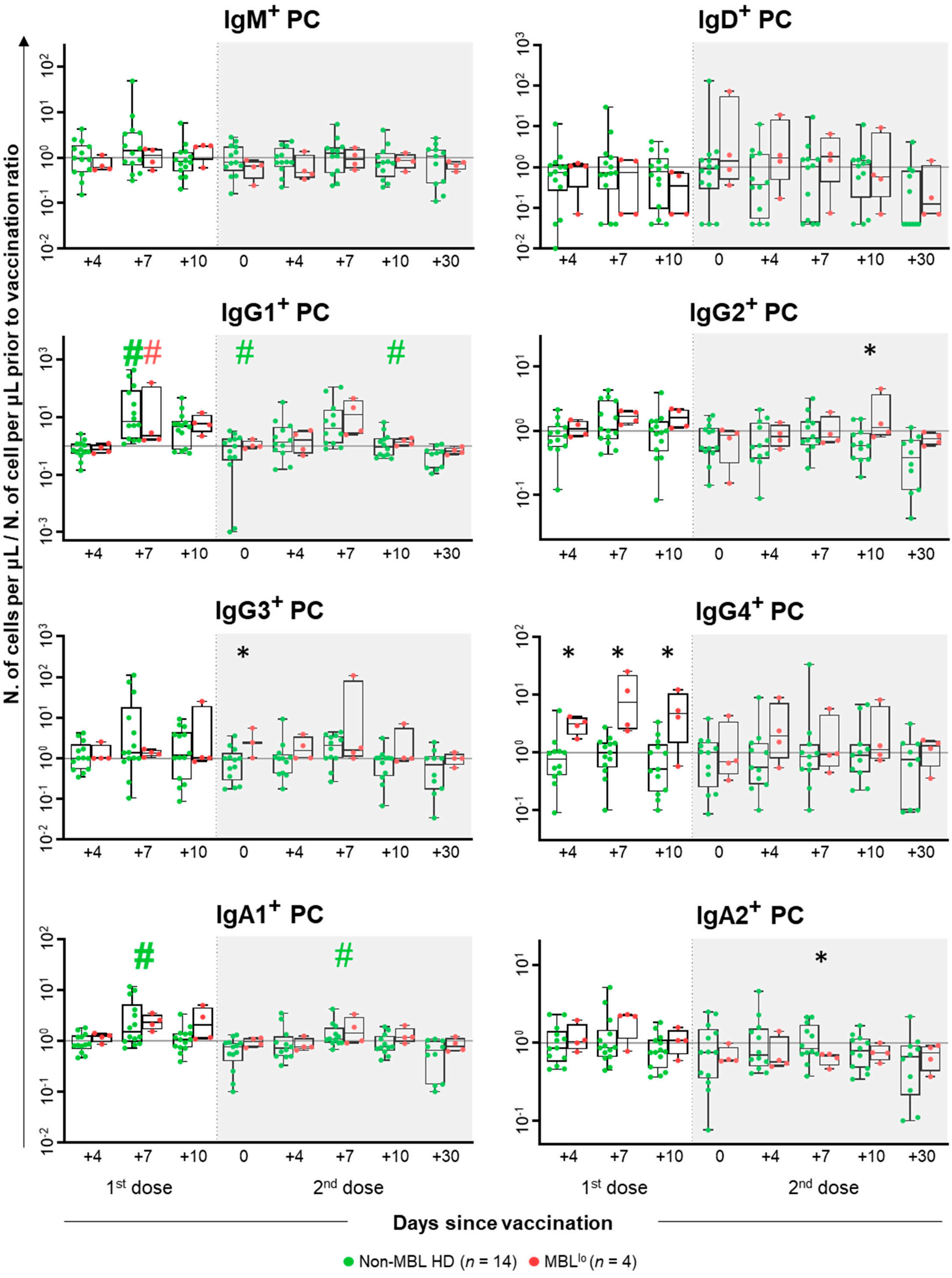
IgM+ PC
IgD+ PC
IgG1+ PC
IgG2+ PC
IgG3+ PC
IgG4+ PC
IgA1+ PC
IgA2+ PC
N. of cells per µL / N. of cell per µL prior to vaccination ratio
+4
+7
+10
0
+30
1st dose
2nd dose
Days since vaccination
Non-MBL HD (n = 14)
MBLlo (n = 4)

Supplementary Figure 20. Longitudinal kinetics of plasma cell subsets defined by their IgH isotype and subclass expression profile in blood of MBL[lo] vs. non-MBL HD after SARS-CoV-2 vaccination (independently of the SARS-CoV-2 infection status before vaccination). Data expressed as the ratio (considering 0 as no change vs. pre-vaccination time-point) between the absolute cell count/µL of each cell population and the cell count detected for the same cell population at baseline, prior to vaccination. Subjects without MBL (green dots) and with MBL[lo] (red dots) with data available for all time points analyzed, are grouped according to the time from the administration of the vaccine. Notched boxes represent 25th and 75th percentile values (IQR), whereas the line in the middle corresponds to median values, and whiskers represent the maximum and minimum values observed for each group. *Statistically significant differences ($p \leq 0.05$) between SARS-CoV-2 naive vs. previously infected non-MBL HD; #Statistically significant differences ($p \leq 0.05$) between the time-point analyzed and the previous one for each (color-coded) group of individuals. Hashtags (#) depicted in bold refer to statistically significant differences when considering a (more stringent) FDR <5%. Abbreviations: HD, healthy donors; Ig, immunoglobulin; IQR, interquartile range; MBL, monoclonal B-cell lymphocytosis; PC, plasma cells.

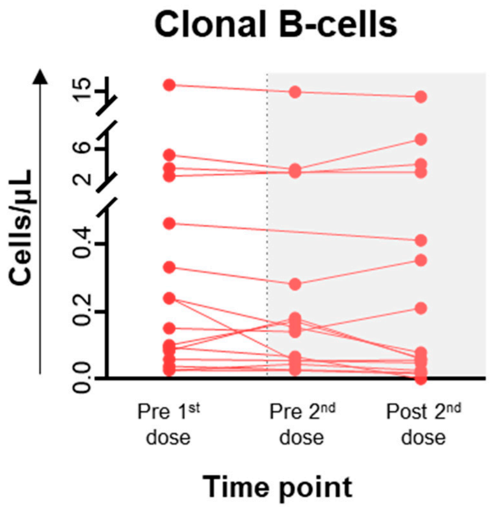

Supplementary Figure 21. Kinetics of clonal B-cells in blood of MBL[lo] individuals after SARS-CoV-2 vaccination. Data expressed as absolute cell counts/µL for each individual cell population analyzed. Abbreviations: IQR, interquartile range; MBL, monoclonal B-cell lymphocytosis.
